# A stromal lineage maintains crypt structure and villus homeostasis in the intestinal stem cell niche

**DOI:** 10.1186/s12915-023-01667-2

**Published:** 2023-08-08

**Authors:** Jinnan Xiang, Jigang Guo, Shaoyang Zhang, Hongguang Wu, Ye-Guang Chen, Junping Wang, Baojie Li, Huijuan Liu

**Affiliations:** 1 The Bio-X Institutes, Shanghai Jiao Tong University, Shanghai, 200024 China; 2 State Key Laboratory of Membrane Biology, Tsinghua-Peking Center for Life Sciences, School of Life Sciences, Tsinghua University, Beijing, 100084 China; 3 Chongqing Engineering Research Center for Nanomedicine, College of Preventive Medicine, Third Military Medical University, Chongqing, China

**Keywords:** Mesenchymal, Niche, ISC, Wntless, Paneth cell, Inflammation

## Abstract

**Background:**

The nutrient-absorbing villi of small intestines are renewed and repaired by intestinal stem cells (ISCs), which reside in a well-organized crypt structure. Genetic studies have shown that Wnt molecules secreted by telocytes, *Gli1*^+^ stromal cells, and epithelial cells are required for ISC proliferation and villus homeostasis. Intestinal stromal cells are heterogeneous and single-cell profiling has divided them into telocytes/subepithelial myofibroblasts, myocytes, pericytes, trophocytes, and *Pdgfra*^*low*^ stromal cells. Yet, the niche function of these stromal populations remains incompletely understood.

**Results:**

We show here that a *Twist2* stromal lineage, which constitutes the *Pdgfra*^*low*^ stromal cell and trophocyte subpopulations, maintains the crypt structure to provide an inflammation-restricting niche for regenerating ISCs. Ablating *Twist2* lineage cells or deletion of one *Wntless* allele in these cells disturbs the crypt structure and impairs villus homeostasis. Upon radiation, *Wntless* haplo-deficiency caused decreased production of anti-microbial peptides and increased inflammation, leading to defective ISC proliferation and crypt regeneration, which were partially rescued by eradication of commensal bacteria. In addition, we show that Wnts secreted by *Acta2*^+^ subpopulations also play a role in crypt regeneration but not homeostasis.

**Conclusions:**

These findings suggest that ISCs may require different niches for villus homeostasis and regeneration and that the *Twist2* lineage cells may help to maintain a microbe-restricted environment to allow ISC-mediated crypt regeneration.

**Supplementary Information:**

The online version contains supplementary material available at 10.1186/s12915-023-01667-2.

## Background

The small intestinal villi, tissue responsible for nutrient absorption, are renewed every 3–5 days by *Lgr5*^+^ intestinal stem cells (ISCs) located at the crypt base [1]. ISCs and Paneth cells form the stem cell zone and shape the crypt architecture [2–4]. Paneth cells are derived from ISCs and located at the base of crypts, where they secrete anti-microbial peptides (AMPs) including defensin/cryptdin/CRS molecules to fend off the microbiota (at the order of 10^2^–10^7^ cells/g in small intestines in human) [5, 6]. The villi are easily damaged by food-borne pathogens, carcinogenic agents, or radiation, which are repaired by ISCs or stem cells derived from committed or differentiated cells [7, 8]. Some injuries disrupt the physical barrier and result in microbial invasion and inflammation, which present an extra threat to ISCs [9]. The ISC activities in homeostasis and regeneration are regulated by niche cells, e.g., Paneth cells and stromal cells, which secrete molecules such as R-Spondin, Wnts, and BMPs [10–13]. Although the depletion of Paneth cells does not affect villus homeostasis [14, 15], there is evidence that both epithelial cells and stromal cells cooperate to support ISCs [16–19].

Recent studies suggest that intestinal stromal cells/myofibroblasts provide Wnt molecules to support ISC proliferation and organiod growth [20–23]. These cells are likely derived from the mesenchyme of mesoderm during development and can be divided into several subpopulations based on multiple single-cell RNA-sequencing (scRNA-seq) data, telocytes/subepithelial myofibroblasts, myocytes, pericytes, trophocytes, and *Pdgfra*^*low*^ stromal cells [24–27]. The intestinal stromal cells/myofibroblasts can also be genetically marked by *Pdgfra*, *Gli1*, *Foxl1*, *Ng2*, or *Gremlin 1* [13, 16, 22, 24, 25, 28]. Deletion of *Porcn*, which encodes an enzyme required for palmitoylation and secretion of Wnts, in *Pdgfra*^+^ stromal cells leads to neonatal lethality with decreases in villus size and density [22]. Inducible deletion of *Porcn* in *Foxl1*^+^ telocytes, gigantic cells that each encompasses dozens of villus/crypt cells, leads to villus and crypt collapse within 3 days of induction [28]. Intriguingly, blockade of Wnt secretion in all cells by ablation of Wntless (*Wls*) (using *Rosa-CreERT; Wls*^*f/f*^ mice) or killing stromal cells marked by *Gli1*, which is required for *Foxl1* expression [29], takes 10 days to show obvious defects [24, 30]. One explanation for these conflicting results is that Porcn and Wntless may not have identical functions [31–33]. Intriguingly, expression of the genetic markers is not restricted to a specific stromal/myofibroblast subpopulation. For example, *Pdgfra* is expressed in telocytes, trophocytes, and *Pdgfra*^*low*^ stromal cells, *Gli1* is expressed in some telocytes, myocytes, trophocytes, and *Pdgfra*^*low*^ stromal cells, *Foxl1* is expressed in telocytes, myocytes, and some epithelial cells, and *Acta2* (encoding αSMA) is expressed in telocytes, myocytes, and pericytes [34, 35]. Nevertheless, the relationships of genetically-marked cells and the subpopulations defined by scRNA-seq and the functions of these subpopulations warrant further investigation [36].

Herein, we tested whether genetic markers for bone marrow mesenchymal stem/stromal cells (BM-MSCs), *Prrx1*, *Twist2*, *Nestin*, and *Acta2*, labeled intestinal stromal cells [37]. We show that the *Twist2* lineage cells represent the largest among the four lineages examined. Single-cell profiling suggests that *Twsit2* is expressed in portions of the trophocyte and *Pdgfra*^*low*^ stromal cell subpopulations, but not *Acta2*^+^ subpopulations. In contrast to the findings that deletion of *Wls* in *Ng2*- or *Gli1*-marked stromal cells do not affect the intestinal villus [16, 25], ablation of even one *Wls* allele in *Twist2* lineage cells, but not *Prrx1*, *Nestin*, or *Acta2* lineage cells, disturbed the crypt structure and villus homeostasis. Moreover, upon IR-induced injury, *Wls* haplo-deficiency resulted in decreases in the number of Paneth cells and the production of antimicrobial proteins, accompanied by increased inflammation and impaired crypt regeneration. These defects were largely rescued by the eradication of commensal bacteria with antibiotics. In addition, *Acta2*-marked telocytes, myocytes, and pericytes, but not *Prrx1* or *Nestin* lineage cells, play a niche role in crypt regeneration but not homeostasis. This study thus uncovered ISC niche components with previously unidentified functions.

## Results

### Labeling of intestinal stromal subgroups by *Twist2, Prrx1, Nestin* and *Acta2*

**Fig. 1:**
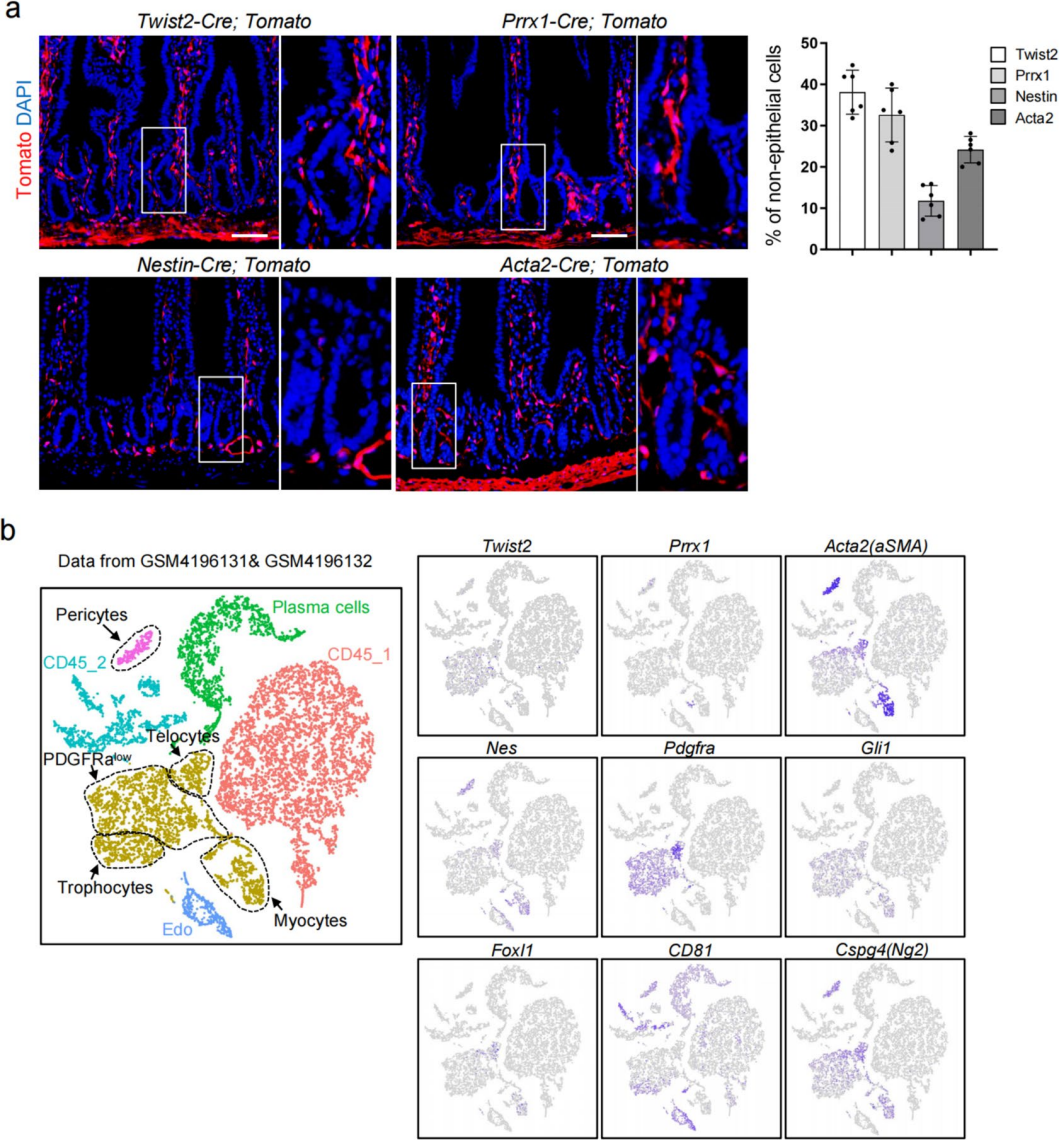
Lineage tracing and scRNA-seq analysis of *Twist2* lineage cells in the intestinal villi. **a** Detection of stromal lineages labeled by *Twist2*, *Prrx1*, or *Nestin*, and *Acta2*^+^ myofibroblasts in the small intestinal villi of adult mice. Right panel: the percentage of *Twist2*, *Prrx1*, and *Nestin* lineage cells and *Acta2* lineage myofibroblasts in non-epithelial (EpCAM^+^) cells. Bar: 50 μm. The calculation was done by counting Tomato^+^ cells on six views per mouse and divided by the number of non-epithelial DAPI^+^ nuclei, *N* = 6. **b** Single-cell transcriptome analysis of small intestinal cells. A scRNA-seq dataset (PMID 32084389) was used to analyze the expression of various stromal/myoblast cell markers

To understand the roles of various intestinal stromal subpopulations in villus homeostasis and regeneration, we tested whether BM-MSC markers, *Prrx1*, *Twist2*, and *Nestin* marked intestinal stromal cells [38–41]. We crossed *ROSA-tdTomato* reporter mice to the Cre mouse lines driven by the promoter of these genes to perform genetic tracing. We showed that *Prrx1*, *Twist2*, and *Nestin* marked 32.6%, 38.1%, and 11.8% of non-epithelial intestinal villus cells, respectively in adult mice, and some of these cells were located at the base of the crypts (Fig. 1a). We also included *Acta2* lineage cells for comparison since the roles of myofibroblasts remain not well understood in crypt regeneration compared to homeostasis [42, 43]. We found that *Acta2* marked 24.2% of non-epithelial intestinal cells in *Acta2-Cre;Rosa-tdTomato* mice (Fig. 1a). In these lineage tracing experiments, none of the EpCAM^+^ epithelial cells were Tomato^+^ (data not shown). These results indicate that *Twist2* marks the largest portion of intestinal stromal/myofibroblast cells (38.1%) among the 4 marker genes.

Single-cell RNA-seq analyses suggest that intestinal stromal cells are heterogeneous and can be divided into telocytes/subepithelial myofibroblasts, myocytes, pericytes, trophocytes, and *Pdgfra*^*low*^ stromal cells (reviewed in [34]), although they may have redundant functions in controlling ISC activities [24, 25]. We re-analyzed the scRNA-seq data of intestinal cells and found that intestinal stromal/myofibroblast cells indeed formed the 5 subpopulations (Fig. 1b and additional file: Fig. S1a) [25]. Moreover, *Twist2*, *Prrx1*, *Acta2*, and *Nestin* were expressed only in stromal/myofibroblast cells but not in immune cells and these 4 marker genes showed distinct expression patterns (Fig. 1b and additional file: Fig. S1a). Notably, *Twist2* was mainly expressed in portions of trophocytes (*Pdgfra*^*low*^*CD81*^+^) and *Pdgfra*^*low*^*CD81*^*−*^ stromal cells, but not much in telocytes/subepithelial myofibroblasts, myocytes, or pericytes (Fig. 1b). *Prrx1* was expressed in a portion of myocytes, *Acta2* was expressed in telocytes/subepithelial myofibroblasts, myocytes, and pericytes, and *Nestin* was expressed in portions of *Acta2*-expressing subpopulations (Fig. 1b), consistent with the observation that *Nestin* marked fewer cells than *Acta2* in tracing experiments. We also compared the expression of *Gli1* and *Twist2* cells in trophocytes (*Pdgfra*^*low*^*CD81*^+^) and *Pdgfra*^*low*^*CD81*^*−*^ stromal cells and found that their expression showed minimal overlapping (Additional file: Fig. S1b). We also compared the expression of surface markers on the intestinal *Twist2* and *Gli1* lineage cells and found that *Twist2* lineage cells were positive for CD29 and CD106 but negative for CD44, CD73, CD105, and Sca-1, while *Gli1* lineage cells in *Gli1-CreERT;Rosa-tdTomato* mice were positive for CD29, CD44, and CD73 but negative for CD105, CD106, and Sca1 (Additional file: Fig. S2). Overall, these data suggest that the *Twist2* lineage cells are distinct from cells marked by *Prrx1*, *Nestin*, *Acta2*, or *Gli1*. Note that we used a *Twist2-Cre* line in this study which cumulatively marked the Twist2 lineage cells. However, the labeling of only 38.1% of intestinal stromal/myofibroblast cells and restricted expression pattern of *Twist2* in trophocyte and *Pdgfra*^*low*^ subpopulations suggest that the *Twist2* lineage may not contribute to telocyte, pericyte, and myocyte subpopulations. Certainly, further verification will need CreERT lines for *Twist2*, *Acta2*, *Prrx1*, and *Nestin*.

### Depletion of the *Twist2* lineage disrupts crypt structure and villus homeostasis

**Fig. 2:**
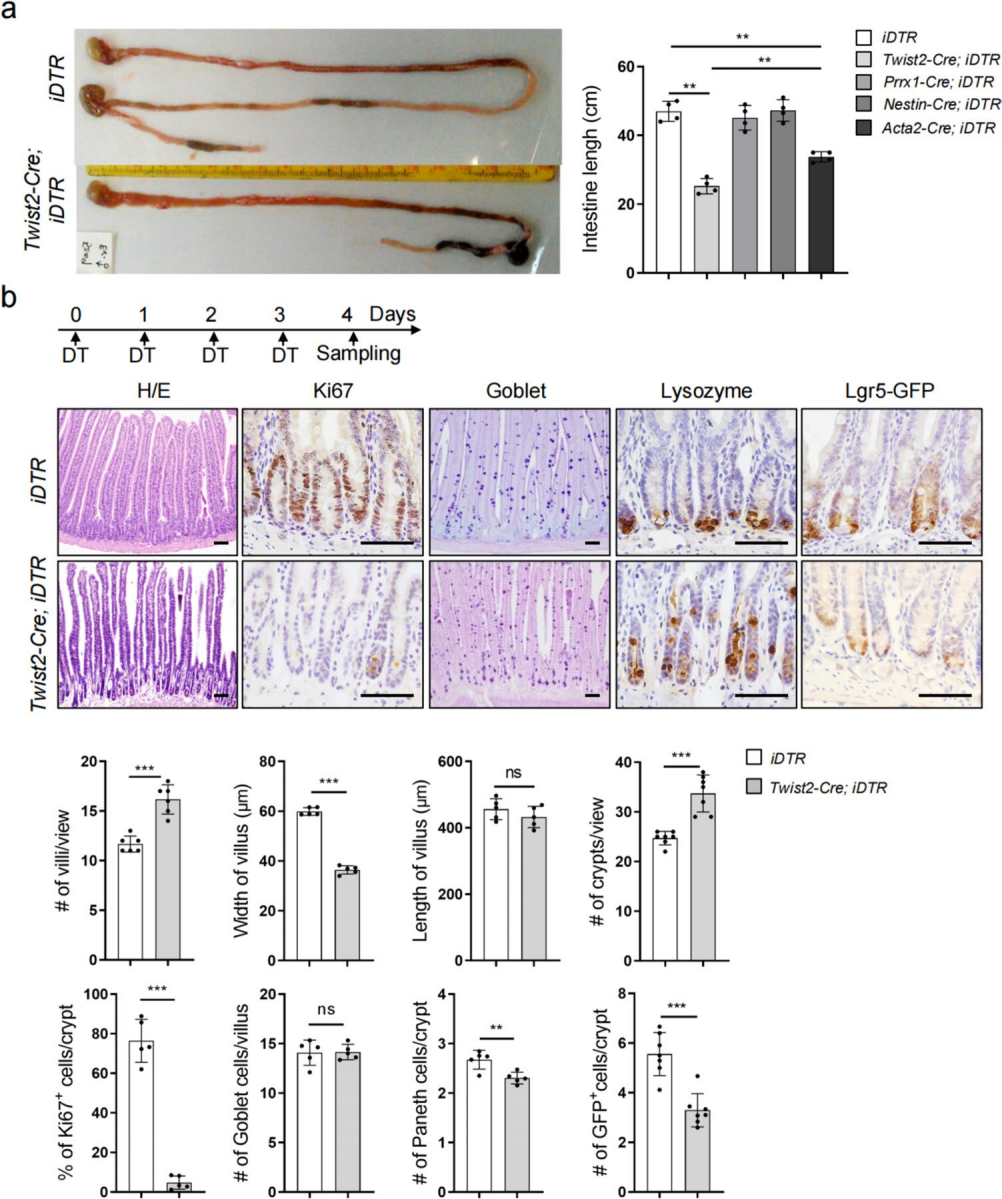
Depletion of *Twist2* lineage cells impaired villus homeostasis. **a** Depletion of the *Twist2* or *Acta2* lineage, but not the *Prrx1* or *Nestin* lineage, led to shortening of the GI tracts. Right panel: quantitation data of the intestine length. *N* = 4. See additional file: Fig. S3e for details. **b** H/E, Ki67, goblet, lysozyme, and GFP staining of normal and *Twist2* cell-depleted mouse intestinal villi. Also, see Fig. S3g for control. Bar: 50 μm. Bottom panels: quantitation data. **P < 0.01 and ***P < 0.001. ns: not significant. *N* = 5. *Lgr5-GFP*: *Lgr5-EGFP-IRES-CreERT2*

To determine which stromal populations(s) play a role in villus homeostasis, we used an inducible diphtheria toxin receptor system (*ROSA26-iDTR* mouse) to transiently deplete each of these populations [44]. Daily injection of diphtheria toxin (DT) for 4 days led to a great reduction in the numbers of BM-MSCs in *Prrx1-Cre; iDTR* mice, *Nestin-Cre; iDTR* and *Twist2-Cre; iDTR* mice (Additional file: Fig. S3a and b), and immunostaining showed that αSMA^+^ cells were reduced in the intestines of *Acta2-Cre; iDTR* mice (Additional file: Fig. S3c). These mouse lines showed increased mortality compared to DT-treated control mice and survived for different periods of time (Additional file: Fig. S3d). Depletion of the *Twist2* or *Acta2* lineage for 4 days led to the shortening of small intestinal tracts while depletion of *Prrx1* or *Nestin* lineage for 7 days did not produce obvious phenotypes (Fig. 2a and Additional file: Fig. S3e). Depletion of the *Twist2* lineage caused red and swollen intestines, indicative of inflammation.

Depletion of the *Twist2* lineage led to thinned villi (Fig. 2b). The morphology of the crypt was altered compared to DT-treated *ROSA-iDTR* mice or PBS-treated *Twist2-Cre; iDTR* mice (Fig. 2b and Additional file: Fig. S3f). The crypts were thinner with the boundary between crypts and villus becoming indistinct, accompanied by decreases in Ki67-positive proliferating cells and the number of Paneth cells with some Paneth signals detected at the upper part of the crypts (Fig. 2b). Furthermore, depletion of *Twist2* lineage cells led to a decrease in the number of *Lgr5*^+^ ISCs, with some of *Lgr5*^+^ ISCs being dislocated (Fig. 2b). Thus, depletion of *Twist2* lineage cells altered the structure of the crypts. Depletion of *Acta2* lineage cells, but not *Prrx1* cells, also caused a decrease in villus size and cell proliferation (Additional file: Fig. S3g), although to a much lesser extent than depletion of *Twist2* cells.

We then focused on the *Twist2* lineage cells. The *Twist2-Cre* mouse line had the Cre cassette inserted into the coding sequence, which disrupted this gene. In this study, we used the heterozygous *Twist2-Cre* mice to perform genetic tracing or gene ablation. The heterozygous *Twist2-Cre* mice showed normal villus structure, normal cell proliferation, and differentiation of goblet and Paneth cells (Additional file: Fig. S4a), suggesting that *Twist2-Cre* mice behaved like wild-type mice and can be safely used to trace *Twist2* lineage cells or to ablate *Twist2* lineage cells. Single-cell profiling revealed that *Twist2* is not expressed in CD45^+^ immune cells or CD31^+^ endothelial cells (Additional file: Fig. S1a). Further, our previous genetic tracing and bone marrow graft assays revealed that *Twist2-Cre;Rosa-Tomato* mice did not show labeling of immune cells under normal conditions or in DSS-induced colitis model mice [45].

We tested the potential involvement of *Twist2* lineage cells in crypt regeneration after IR-induced damage. IR at 6.5 Gy induces modest crypt and villus injury in normal adult mice, which can be repaired quickly, as previously reported [46]. We found that *Twist2* lineage depletion mice could survive less than 4 days post IR and they showed more severe damage than control mice, manifested by decreases in the numbers of villi, crypts, proliferating cells, Paneth cells, and ISCs (Additional file: Fig. S4b). These results indicate that *Twist2* lineage cells play a role in crypt regeneration as well.

### *Twist2* lineage-secreted Wnts are needed for crypt structure integrity and homeostasis

**Fig. 3:**
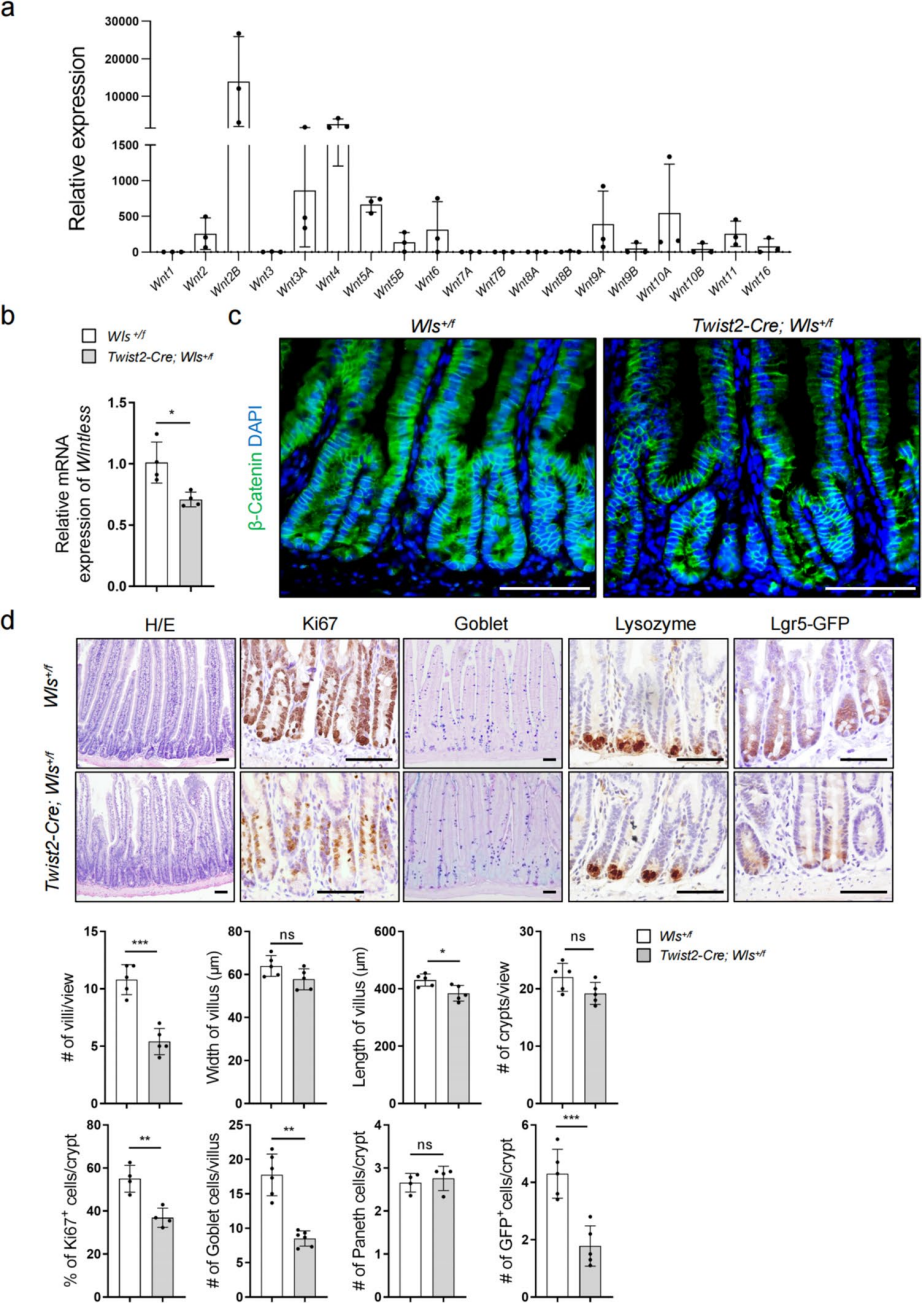
Intestinal *Twist2* cell-secreted Wnts were critical for villus homeostasis and crypt structure maintenance. **a** Expression of Wnt molecules in *Twist2* intestinal stromal cells. Intestinal Tomato^+^ stromal cells were sorted from *Twist2-Cre; Tomato* mice and collected for RNA isolation and qPCR analysis. The Value of *Wnt1* was set at 1.0. *N* = 3. **b** Quantitative PCR results showed that stromal cells (Tomato^+^) of the small intestine of *Twist2-Cre;Tomato;Wls*^+*/f*^ mice showed a decrease in the mRNA levels of *Wls* compared to control mice. *N* = 4. **c** Representative immunofluorescent staining results for β-Catenin in the intestinal villi of control and *Twist2-Cre;Wls*^+*/f*^ mice. Bar: 50 μm. The intestine sections were stained with anti-β-Catenin antibodies and DAPI. β-Catenin immunofluorescent staining results without DAPI is shown in additional file: Fig. S6. **d** H/E, Ki67, goblet, Paneth cells, and GFP staining of intestinal villi of control and *Twist2-Cre;Wls*.^+*/f*^ mice. Bar: 50 μm. Bottom panels: quantitation data. *P < 0.05, **P < 0.01, and ***P < 0.001. *N* = 4 (% of Ki67 + cells/crypt and # of Paneth cells/crypt). *N* = 6 (# of Goblet cells/villus). Others *N* = 5

The niche cells provide signaling molecules including Wnts to support ISC proliferation [22, 28]. Following up on the lead obtained from our cell ablation experiments, we analyzed the expression of Wnt molecules in *Twist2* lineage stromal cells and found that these cells expressed some Wnt molecules, especially *Wnt 2B*, *3A*, *4*, and *5A* (Fig. 3a). scRNA-seq analysis also showed that *Twist2*^+^ subpopulations expressed high levels of *Wnt2B*, *4*, and *5A*, as well as *Rspo1-3* (Additional file: Fig. S5). Both Wnt and R-Spondin (encoded by *Rspo*) molecules are critical niche signals for ISCs. However, the *Wnt* expression pattern did not entirely overlap with the intestinal mesenchymal mixtures [20], consistent with the notion that the *Twist2* lineage does not represent all intestine stromal cells.

To determine the significance of Wnt molecules secreted by *Twist2* lineage cells, we ablated *Wls*, which encodes a sorting receptor for Wnt secretion [47], by crossing *Twist2-Cre* mouse to *Wls*^*f/f*^ mouse that also carries a *Lgr5-GFP* allele to label ISCs. Homozygous deletion of *Wls* in *Twist2* lineage cells led to embryonic lethality at E18.5 whereas *Wls* heterozygous mice were viable. We found that deletion of even one *Wls* allele in *Twist2* lineage led to a reduction of *Wls* expression and a decrease in β-Catenin, a downstream target stabilized by Wnt activation (Fig. 3b, c, and Additional file: Fig. S6). Note that *Wls* deficiency also led to a decrease of β-Catenin in the enterocytes of the villi (Fig. 3c), suggesting that Wnt molecules secreted by *Twist2* lineage cells located at the upper part of the villi may also play a role. Phenotypically, the mutant mice showed significant decreases in the number and size of villi and the numbers of proliferating cells and goblet cells (Fig. 3d), and a portion of the crypts appeared thinner with the boundary between crypts and villi being obscure (Fig. 3d). Moreover, the number of *Lgr5*^+^ ISCs was reduced and some ISCs showed mis-localization (Fig. 3d). Thus, deletion of one *Wls* allele in *Twist2* lineage disrupts villus homeostasis as well as the crypt structure.

Ablation of *Wls* in the *Prrx1* lineage led to embryonic lethality while the heterozygous mice were viable. *Prrx1-Cre; Wls*^+*/f*^ mice showed normal levels of β-Catenin in enterocytes and unaltered number and size of villi or crypts (Additional file: Fig. S7a and b). *Acta2-Cre; Wls*^*f/f*^ mice, which were viable, showed no obvious defects in villus and crypt structure (Additional file: Fig. S7a and b). One explanation is that the Prrx1 or Acta2 lineage stromal cells synthesize much less Wnt molecules than Twist2 lineage cells, which warrants further investigation. These results highlight the importance of *Twist2* cells-secreted Wnts in villus homeostasis.

### Wnts secreted by *Twist2* lineage are required for villus and crypt development

**Fig. 4:**
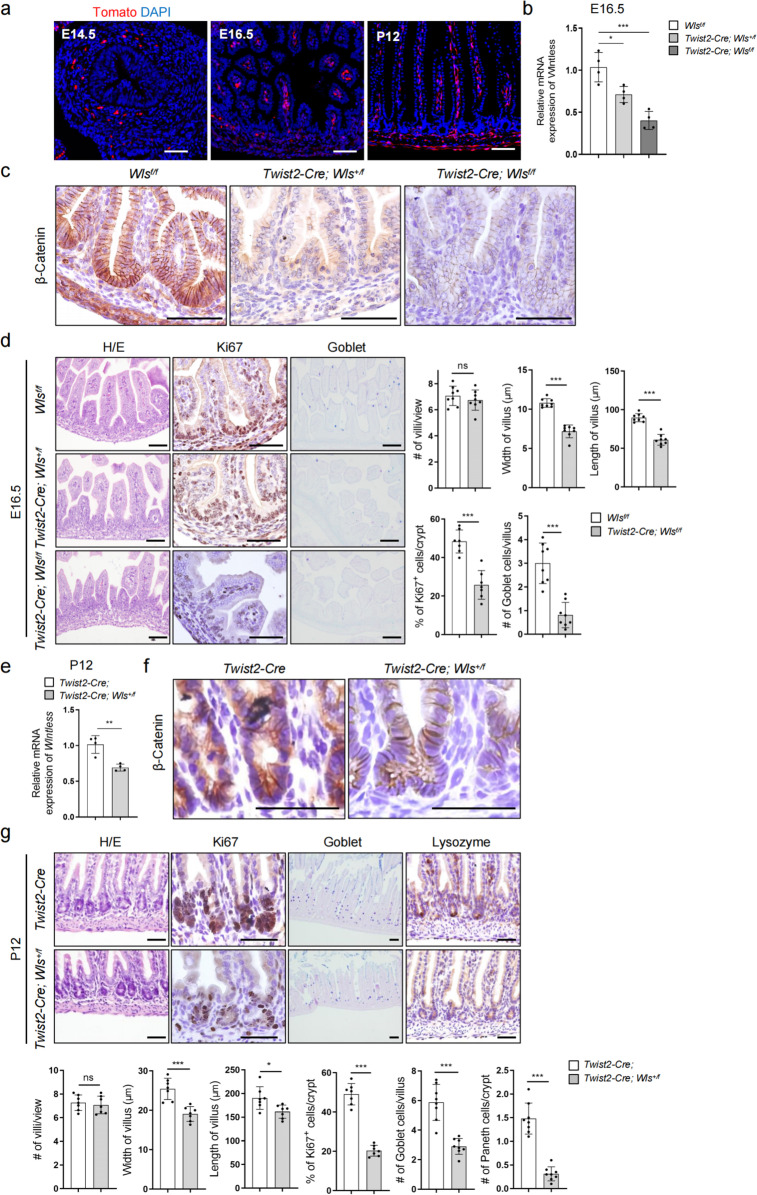
*Twist2* cell-secreted Wnts were critical for villus and crypt development. **a** Detection of *Twist2* lineage cells in E14.5, E16.5, and p12 *Twist2-Cre;Tomato* mouse intestines. Bar: 50 μm. **b** Quantitative PCR results of the intestinal stromal cells (Tomato^+^) of E16.5 control, *Twist2-Cre;Wls*^*f/f*^, and *Twist2-Cre;Wls*^+*/f*^ embryos. *N* = 4. **c** Representative immunohistochemical staining results for β-Catenin in the intestinal villi of E16.5 control, *Twist2-Cre;Wls*^*f/f*^, and *Twist2-Cre;Wls*^+*/f*^ embryos. Bar: 50 μm. Brown color indicated β-Catenin signals. **d** H/E, Ki67, and goblet staining of intestinal villi of E16.5 control, *Twist2-Cre;Wls*^*f/f*^, and *Twist2-Cre;Wls*^+*/f*^ embryos. Bar: 50 μm. Bottom panels: quantitation data. ***P < 0.001. *N* = 8 or *N* = 7 (Ki67). **e** Quantitative PCR results of the intestinal stromal cells (Tomato^+^) of P12 control and *Twist2-Cre;Wls*^+*/f*^ pups. *N* = 4. Brown color indicated β-Catenin signals. **f** Representative immunohistochemical staining results for β-Catenin in the intestinal villi of P12 control and *Twist2-Cre;Wls*^+*/f*^ pups. Bar: 50 μm. **g** H/E, Ki67, goblet, and Paneth cell staining of intestinal villi of p12 control and *Twist2-Cre; Wls*.^*f/f*^ embryos. Bar: 50 μm. Bottom panels: quantitation data. *P < 0.1, **P < 0.01, and ***P < 0.001. *N* = 7

Mouse intestinal villi first emerge at E14.5 [48]. *Twist2* lineage cells were detected in intestinal structures as early as E14.5 (Fig. 4a). An early study showed that *Twist2-Cre; Wls*^*f/f*^ mice were lethal at E13.5 and the in vitro cultured small intestine showed a defect in epithelial cell proliferation [49]. Our *Twist2-Cre; Wls*^*f/f*^ mice died at E18.5, likely due to different genetic background. We analyzed the intestine of E16.5 embryos and found that deletion of two *Wls* alleles in *Twist2* lineage cells led to decreases in *Wls* expression and β-Catenin (Fig. 4b and c), which were associated with smaller villi and decreases in proliferating cells and goblet cells (Fig. 4d). Moreover, the E16.5 *Twist2-Cre; Wls*^+*/f*^ embryos also showed smaller villi and decreases in proliferating cells and goblet cells (Fig. 4b–d). The intestinal crypts are formed around p12 [3, 50]. We found that P12 *Twist2-Cre; Wls*^+*/f*^ mice exhibited decreases in *Wls* expression and β-Catenin (Fig. 4e and f), which were associated with decreases in the crypt size and the numbers of proliferating cells, goblet cells, and Paneth cells (Fig. 4g). Thus, Wnt molecules secreted by the *Twist2* lineage play important roles in villus development and crypt formation.

### *Twist2* stromal cell-secreted Wnts are needed for crypt regeneration

**Fig. 5:**
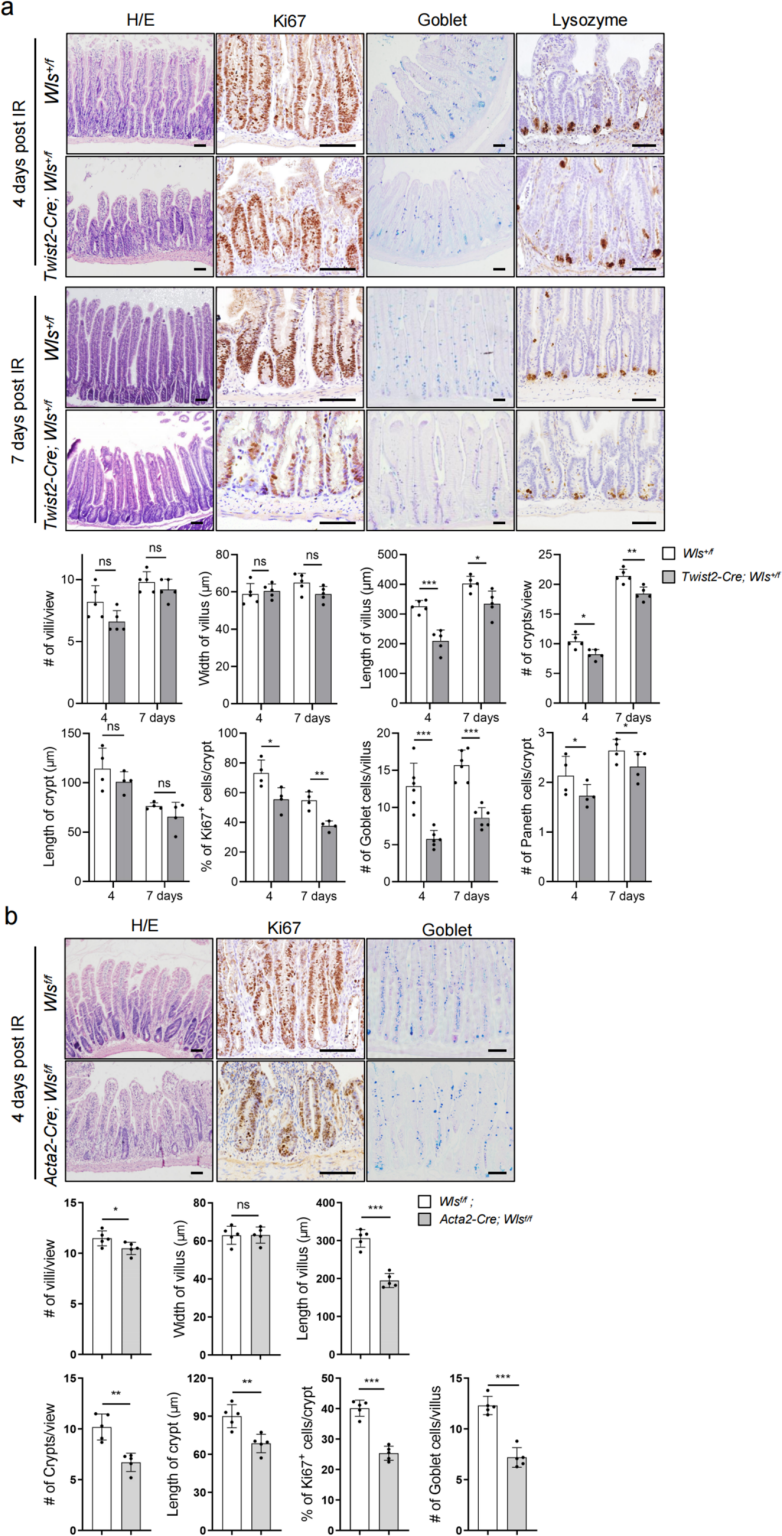
Intestinal *Twist2* cell-secreted Wnts were critical for crypt regeneration. **a** H/E, Ki67, goblet, and Paneth cell staining of intestinal villi of control and *Twist2-Cre;Wls*^+*/f*^ mice 4 or 7 days after IR (6.5 Gy). Bar: 50 μm. Right panels: quantitation data. *P < 0.05, **P < 0.01, and ***P < 0.001. *N* = 4. **b** *Acta2-Cre; Wls*^*f/f*^ mice showed a defect in crypt regeneration 4 days after IR. H/E, Ki67, and goblet cell staining of intestinal villi of control and *Acta2-Cre; Wls*.^*f/f*^ mice 4 days after IR (6.5 Gy). Bar: 50 μm. Right panels: quantitation data. *P < 0.05, **P < 0.01, and***P < 0.001. *N* = 5

Moreover, *Twist2-Cre;Wls*^+*/f*^ mice showed defects in villus/crypt regeneration, manifested by decreases in the size of villi, the number of crypts, the number of proliferating cells, and the numbers of goblet cells and Paneth cells at day 4 or 7 following IR (Fig. 5a). The difference in the villi and crypts between the mutant and control mice at day 7 post IR was much greater than that without IR (Fig. 5a), suggesting that the repair process was impaired in *Twist2-Cre;Wls*^+*/f*^ mice.

*Prrx1-Cre;Wls*^+*/f*^ mice did not show obvious defects in crypt regeneration following IR (Additional file: Fig. S8). However, the *Acta2-Cre;Wls*^*f/f*^ mice could survive only 4–5 days after IR and villus regeneration was compromised and the number and size of crypts and the numbers of proliferating cells and goblet cells were reduced (Fig. 5b). Thus, Wnt molecules secreted by *Acta2*-marked cells play critical roles in crypt regeneration but not in homeostasis.

### Synthesis of AMPs and inflammatory cytokines during normal crypt regeneration

Paneth cells mainly exist in the small intestines, where they secrete anti-microbial peptides to fend off commensal bacteria. A recent study showed that Paneth cells may dedifferentiate to ISCs following IR [8]. We found that after IR, apoptosis occurred to enterocytes and the number of Paneth cells went down but recovered later (Additional file: Fig. S9a). Interestingly, we observed a peak expression of AMPs including *Crypt1*, *Crypt4*, *Plys*, *CRS1C*, and *CRS4C*, but not *mLys*, *Reg3g*, *TCF4*, or *MMP7* at day 2 post IR, which might be attributable to surviving Paneth or other cells (Additional file: Fig. S9b). We also found an increase in the levels of *IL1β*, *IL6*, *IL17α*, and *IL22* at day 3 post IR, which went down at day 4, while the levels of *TNFα* and *IFNγ* were modestly increased, which persisted through the regeneration process (Additional file: Fig. S9c), suggestive of inflammation. AMPs secreted by Paneth cells may help restrict bacterial propagation and generate an environment suitable for crypt regeneration.

### Decreased AMPs and increased inflammation in regenerating intestines of *Twist2-Cre; Wls*^+/f^ mice

**Fig. 6:**
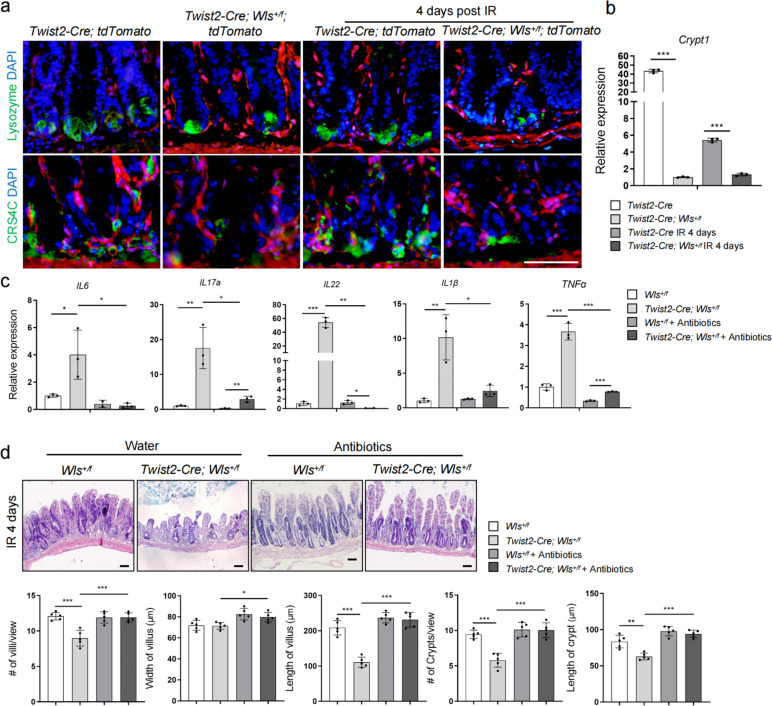
Crypt regeneration defects in *Twist2-Cre; Wls*^+*/f*^ mice were rescued by antibiotics treatment. **a** Decreases in the number of Paneth cells and expression of AMPs in regenerating villi of *Twist2-Cre; Wls*^+*/f*^ mice. Bar: 50 μm. **b** qPCR analysis showed reduced expression of *Crypt4* in the villi of villi of *Twist2-Cre;Wls*^+*/f*^ mice. The basal levels in *Twist2-Cre; Wls*^+*/f*^ mouse intestines were set at 1.0. ***P < 0.001. *N* = 4. **c** IR-induced production of inflammatory cytokines in the intestine was enhanced in *Twist2-Cre; Wls*^+*/f*^ mice, which was rescued by antibiotic treatment. The basal levels of cytokines in normal mouse intestines were set at 1.0. *P < 0.05, **P < 0.01, and ***P < 0.001. *N* = 3. **d** Crypt regeneration defects in *Twist2-Cre; Wls*.^+*/f*^ mice were rescued by antibiotics treatment. Bar: 50 μm. Bottom panels: quantitation data. *P < 0.05, **P < 0.01, and ***P < 0.001. *N* = 5

In accordance with a decrease in Paneth cells in the intestine of *Twist2-Cre; Wls*^+*/f*^ mice following IR (Fig. 5a), immunostaining revealed that the signals for anti-microbial proteins Lysozyme and CRCS4C were reduced (Fig. 6a). Expression of *Crypt1* was also reduced in *Twist2-Cre; Wls*^+*/f*^ mice, under homeostatic conditions or at day 4 post IR (Fig. 6b). It has been established that loss or reduction of Paneth cells or AMPs production allows for commensal microbial infection, which led to aggravated intestinal inflammation [51]. We indeed found that IR-induced injury led to an increase in the levels of inflammatory cytokines including *IL6* and *TNFα* in the intestinal samples of *Twist2-Cre; Wls*^+*/f*^ mice compared to control mice (Fig. 6c). Overall, these findings suggest that Wnts secreted by *Twist2* stromal cells may help promote Paneth cell recovery and AMP secretion to contain microbes and inflammation.

### Antibiotics partially rescue villus repair defects in *Twist2-Cre; Wls*^+/f^ mice

Emerging evidence suggests that persistent inflammation impairs tissue stem cells including ISCs [52]. We showed that LPS, a bacterial product, augmented cytokine synthesis and impeded crypt regeneration following IR (Additional file: Fig. S10a and b). We suspect that enhanced intestinal inflammation in *Twist2-Cre; Wls*^+*/f*^ mice is likely caused by the invasion of commensal microbes due to decreases in the number of Paneth cells and the production of AMPs. To verify this, we pretreated these mice with a combination of antibiotics to eliminate commensal bacteria before IR. We found that the levels of inflammatory cytokines were suppressed in the intestines of antibiotics-treated *Twist2-Cre; Wls*^+*/f*^ mice (Fig. 6c). Moreover, the crypt regeneration defects in *Twist2-Cre; Wls*^+*/f*^ mice were partially rescued as well (Fig. 6d). These results collectively suggest that the *Twist2* lineage-produced Wnt molecules help establish an inflammation-restricted environment to facilitate crypt regeneration.

## Discussion

The current study shows that the small intestinal stromal/myofibroblast subpopulations can be genetically marked by *Prrx1*, *Twist2*, and *Nestin* in addition to *Acta2*. Single-cell profiling data suggest that these markers are expressed in different subpopulations of the small intestine. In particular, the *Twist2* lineage cells form the *Pdgfra*^*low*^ stromal cell and trophocyte subpopulations, while *Acta2* lineage cells form the telocyte, myocyte, and pericyte subpopulations. While *Nestin*-expressing cells showed some overlapping with *Acta2*-expressing cells, *Prrx1* is expressed in a portion of the myocytes. Moreover, *Twist2*-expressing cells and *Gli1*-expressing cells in the *Pdgfra*^*low*^ stromal cells and trophocytes show little overlapping. While our findings support the clustering of intestinal stromal/myofibroblast cells [24, 25, 34], and that Twist2-marked cells are different from those marked by Prrx1, Acta2, or Nestin, the exact relation between these 4 subpopulations needs further investigation. Additionally, future studies are needed to reconcile the genetic tracing results and the scRNA-seq results. This may require more specific genetic markers and genetic tracing (at different times) in combination with single-cell profiling of the daughter cells.

We show that *Twist2* stromal cells exhibit important niche activities in ISC-mediated villus homeostasis and regeneration, by secreting Wnt molecules. Moreover, deletion of one allele of *Wls* in *Twist2* lineage cells generates more severe villus defects than depletion of *Wls* in *Gli1*^+^ cells [16]. Killing *Twist2* lineage cells also produces a more severe villlus phenotype than killing intestinal stromal cells marked by *Gremlin1* [24], which was recently shown to be a marker of skeletal stem cells and trophocytes in the intestine [37]. This can be explained that the *Twist2* lineage cells comprise *Pdgfra*^*low*^ stromal cells and trophocytes, both of which contribute to the regulation of ISCs by secretion of Wnt molecules. Comparison of *Ng2*- and *Bapx1*-marked intestinal stromal cells also supports the redundancy of stromal subpopulations in the regulation of ISC activity [25, 35]. Moreover, ablation of *Wls* in *Twist2* lineage cells, but not the *Prrx1*, *Acta2*, or *Nestin* subpopulation, impairs villus homeostasis, highlighting the importance of the *Twist2* lineage cells in this event.

*Twist2* lineage cells appear to maintain the crypt morphology as well. We show that depletion of *Twist2* lineage cells or deletion of one allele of *Wls* from *Twist2* lineage cells alters crypt size and morphology including change in crypt length and the villus-crypt boundary and mislocalization of some ISCs. This is in contrast to telocyte cell ablation mice, telocyte-specific *Porcn* deletion mice, and mice with *Wls* deleted simultaneously in *Gli1* stromal cells and *Villin*^+^ enterocytes [16, 28], which all caused shrinkage and collapse of the villi and crypts without distorting the crypt morphology. In addition, deletion of *Wls* or *Porcn* in *Ng2* or *Gli1* cells does not obviously affect the crypt morphology in small intestines [16, 25]. These findings suggest that Wnts secreted by *Twist2* stromal cells localized in the upper part of the crypt may play a role in maintaining the crypt structure.

Findings of this study support the notion that crypt regeneration requires a more demanding niche. We show that *Twist2* lineage cell-generated Wnts are required for proper formation and localization of Paneth cells, production of AMPs, and restriction of inflammation in injured intestines. Moreover, deletion of *Wls* in *Acta2* lineage cells (telocytes, pericytes, and myocytes) impaired crypt regeneration but not villus homeostasis, this is inconsistent with the finding that deletion of *Porcn* in *Foxl1*^+^ telocytes causes collapse of the villi [28]. One explanation is that Porcn and Wntless may have non-overlapping functions [31–33]. Recent studies have shown that *Ng2*^+^ cells-secreted Wnt molecules play more important roles in crypt regeneration than in homeostasis [25] and that even macrophage-secreted Wnts are required for crypt regeneration [53]. Overall, these findings suggest that under injury conditions, there might be a greater need for Wnt molecules than in homeostasis, which are supplied by multiple cell types and a defect in even one source may impair crypt regeneration [35].

During crypt regeneration, newly formed Paneth cells appear to shape the crypt morphogenesis and secrete anti-microbial peptides [54]. Its importance is highlighted by its role in the pathogenesis of intestinal Crohn’s disease. Notably, Paneth cell recovery and synthesis of anti-microbial peptides require Wnt signaling [3]. Our findings provide evidence that *Twist2* lineage stromal cells provide the Wnt molecules required for the activities of Paneth cells and imply that the interaction between *Twist2* stromal cells and Paneth cells may generate a microbe-restricted and inflammation-restricted environment to support ISCs and crypt regeneration. Certainly, this warrants further investigation.

## Conclusions

While *Prrx1* and *Nestin* lineage BM-MSCs constitute niches for hematopoietic stem cells, we show that *Twist2* lineage stromal cells act as a niche for ISCs during villus homeostatic and regeneration via secretion of Wnt molecules. We also show that *Twist2* stromal cells help control the structure of the crypts and may provide a unique niche for ISCs during regeneration.

## Methods

### Mouse lines and maintenance

All animal work was in compliance with the recommendations in the National Research Council Guide for the Care and Use of Laboratory Animals, with the protocols approved. The *ROSA-iDTR* mouse was generated in Waisman’s laboratory and *Prrx1-Cre*, *Twist2-Cre*, *Nestin-Cre*, *Acta2-Cre*, *Gli1-Cre/ERT*, *Lgr5-GFP-Cre/ERT*, and *ROSA-tdTomato* mouse lines were purchased from The Jackson Laboratory.

To deplete a given type of cells, 2-month-old *iDTR* mice expressing Cre were injected intraperitoneally with 100 ng of DT (Sigma) twice per day for several consecutive days. To irradiate the mice, lead plates were used to protect other parts of the mice, only the exposed belly received radiation with RAD SOURCE RS 2000 irradiator. Antibiotics treatment (ampicillin: 0.5 g/L, vancomycin 0.25 g/L, metronidazole 0.5 g/L, and neomycin: 0.25 g/L) of the mice was started two days before IR and sustained until the mice were sacrificed. LPS (2.5 mg/kg) was given at the same time of IR by intraperitoneal injection.

To generate the villus/crypt regeneration model, adult male mice were irradiated at 6.5 Gy (only to the abdomen region) and the mice were euthanized after different periods of time. The wildtype mice survived well and the body weight was maintained, as previously reported [46].

For antibiotic treatment, the combined antibiotics (ampicillin: 0.5 g/L, vancomycin: 0.25 g/L, formazan nitazole 0.5 g/L, and neomycin: 0.25 g/L) were added to drinking water till the end of the experiment.

### H/E staining and lineage tracing

After the mice were sacrificed, the full intestine was removed and the mesenteric fat was cleaned. The length of the intestine was measured. The upper part of the intestine (1 cm) was removed and fixed in 4% paraformaldehyde overnight. Samples were then dehydrated, embedded in paraffin, sectioned at 4 μm through a longitudinal crevice, and prepared for staining. For the cryostat section, intestine samples were embedded in OCT (Leica, 14,020,108,926) and frozen in liquid nitrogen. Sections were cut at 6 μm in thickness at − 20℃. To trace tomato-positive cells, cryostat sections were washed, counterstained with mixed DAPI & Mounting solution (1:1), and covered glasses for observation (Upright Microscope Nikon ECLIPSE 80i). H/E staining and ALP staining (Fast Blue RR Salt and Naphthol AS-MX phosphate, Sigma) were carried out following the standard protocols.

### Immunofluorescent microscopy

The frozen sections were warmed at room temperature after being taken from the − 20℃ fridge. Samples were washed and permeabilized with Triton X100 (0.1%) in PBS for 30 min, blocked with goat serum (10%) at RT for 1 h, and incubated with primary antibody at 4℃ overnight. The next day, after washing, the samples were incubated with a secondary antibody at 37℃ for 1 h in darkness, counterstained with mixed DAPI & Mounting solution (1:1), and mounted for observation (Nikon ECLIPSE 80i). The following antibodies were used: αSMA (1:100; Sigma; A5228) and lysozyme (1:200; Abcam; ab108508). CRS4C (1:100) was generated in Dr. Junping Wang’s lab.

### Immunohistochemical staining

The paraffin sections were dewaxed and rehydrated. The samples were treated with 3% H_2_O_2_ for 20 min to quench the endogenous peroxidase activity, permeabilized with Triton X100 (0.1%) in PBS for 30 min. Antigen retrieval was performed with heated 1X sodium citrate. The samples were blocked, incubated with primary antibodies and secondary antibodies that were conjugated to horseradish peroxidase (HRP) as described for immunofluorescent staining. Afterwards, the samples were incubated with a DAB stain kit (Boster), washed, and incubated with the SABC solution at 37℃ for 1 h in darkness. The slides were then dehydrated through 2 changes of 95% alcohol and absolute alcohol, cleared in two changes of xylene, and mounted with resin for observation. Antibodies against β-Catenin (1:150; CST; 610153), Ki67 (1:200; Abcam; ab16667), lysozyme (1:200; Abcam; ab108508), and GFP (1:150; CST; 2956S) were used.

### Paneth cell and goblet staining

The paraffin sections were deparaffinized with xylene and rehydrated through graded alcohol to deionized water. The samples were stained in Alcian Blue solution for 30 min, rinsed in running tap water for 5 min, placed in periodic acid solution (Periodic Acid-Schiff (PAS) Kit, Sigma) for 5 min, rinsed in several changes of deionized water, placed in Schiff Reagent for 15 min, and washed in lukewarm tap water for 10 min. The samples were then dehydrated through two changes of 95% alcohol and absolute alcohol, 2 min each, cleared in 2 changes of xylene, and mounted with resin for observation.

### Intestinal stromal cell isolation

For intestinal stromal cell isolation, mouse intestines were cut into 0.5–1 cm pieces. Tissues were rinsed with cold PBS + P/S for 2 min, immersed into 10 ml 1 mM DTT solution (Sigma), and rocked at 200 rpm for 10 min. The tissues were rinsed again for 2 min, incubated in 10 ml 3 mM EDTA/1 mM HEPES Buffer with rocking at 200 rpm for 10 min at 37℃, transferred into new 6 cm plates, and incubated with digestion solution (100U/ml collagenase VIII and 100U/ml DNase I) for 30 min. The samples were pipetted up and down to break up the tissue. The isolated cells were centrifuged with a low speed at 250 g for 5 min and single-cell suspension was obtained using a 70 μm strainer (BD Falcon).

### FACS analysis and cell sorting

The isolated intestinal stromal cells were analyzed for MSC markers using flow cytometry using a mouse MSC marker antibody panel kit (R&D, Catalog Number SC018), following the protocol provided by the manufacturer. Analysis was done using a BD FACSCalibur. For cell sorting, the isolated cells were resuspended with Flow Cytometry Staining Buffer. Flow cytometry was performed using Beckman MoFlo™ XDP high-speed cell sorter.

### RNA isolation and quantitative PCR

Tissue samples or sorted cells were frozen in liquid nitrogen for storage and total RNA was isolated using TRIzol (Invitrogen). RNA was reverse transcribed to cDNA with PrimeScript™ RT reagent Kit (Takara. Code No. RR037A). Quantitative PCR was carried out in triplicate on LightCycler® 96 Real-Time PCR System (Roche) with a FastStart Universal SYBR Green Master (Roche), using the cDNA samples as templates. The primers for each gene of interest were shown in Additional file: Table S1. mRNA was quantified using the 2-ΔΔCt model, where ΔΔCt = ΔCtexperimental − ΔCtcontrol (ΔCt = Ctgene of intererst − Cthousekeeping gene).

### Bone marrow colony forming unit assays

The bone marrow was flushed out with α-MEM culture medium from the femurs and tibiae. Red blood cells were cracked with red blood cell lysis buffer. Single-cell suspensions were prepared by filtration through a 40-μm strainer. 1 × 10^6^ cells bone marrow cells were plated in 3.5 cm dishes and allowed to grow in α-MEM medium containing 10% fetal bovine serum, 1% L-glutamine, and 1% penicillin–streptomycin (Gibco, Invitrogen Corporation, USA). After 3 days, the non-adherent cells were removed and the adherent cells were sub-cultured. Medium was replaced every 3 days. After 7 days, colonies were stained.

### Single-cell RNA analysis

Raw reads were demultiplexed and mapped to the mouse reference genome with the Cell Ranger version 3.0.1 (10X Genomics) pipeline using the default parameters. The generated gene-cell expression matrix was used for subsequent analysis in R version 3.6.1 using Seurat version 3.1.5.

### Quantitation and statistical analyses

To quantitate the cells in the villi, cells on ten views of three sections from at least 3 mice were counted. Data are given as mean and standard error of mean (SEM) of the results from > 3 samples in each experiment. Statistical analysis and plotting were processed with Graphpad Prism5. Differences between the two groups were measured by the Student’s *t*-test. Two-way ANOVA was performed to compare two corresponding data points. *P* < 0.05 is defined as being significantly different. *P < 0.05, **P < 0.01, and ***P < 0.001.

### Supplementary Information

**Additional file 1:** **Fig. S1.** tSNE analysis of intestinal cells of mice. **Fig. S2**. The intestinal Twist2 and Gli1 lineage cells expressed different surface markers. **Fig. S3. **Effects of depletion of Twist2, Prrx1, or Nestin lineage cells on the villi. **Fig. S4. **Depletion of Twist2 lineage cells impaired crypt regeneration. **Fig. S5. **tSNE analysis of Wnt and Rspo expression in intestinal cells. **Fig. S6.** Ablation of one Wls allele led to a decrease in β-Catenin in epithelial cells. **Fig. S7.** Effects of deletion of one copy of Wls in Prrx1 or Acta2 lineages on villus homeostasis. **Fig. S8.** Deletion of one Wls allele in Prrx1 lineage cells did not affect villus regeneration. **Fig. S9.** IR induced expression of inflammation-related cytokines and AMPs. **Fig. S10.** LPS-induced inflammation impaired ISC regeneration. **Table S1.** Primer sequences used for quantitative PCR.

**Additional file 2:** The individual data values for Figs. 1-6 and S3, 4, 7, 8, 9, 10.

## Data Availability

The mouse scRNA-seq data (GSM4196131 and GSM4196132) were analyzed [24, 55, 56]. All other data generated or analyzed during this study are included in this published article and its additional files. Individual data values are provided in Additional File 2.

## Abbreviation

ISCs: Intestinal stem cells
AMPs: Anti-microbial peptides
scRNA-seq: Single-cell RNA-sequencing
Wls: Wntless
BM-MSCs: Bone marrow mesenchymal stem/stromal cells

## References

[1] Patel KK, Stappenbeck TS (2013). Autophagy and Intestinal homeostasis. Annu Rev Physiol.

[2] Bjerknes M, Cheng H (1981). The stem-cell zone of the small intestinal epithelium. I. Evidence from Paneth cells in the adult mouse. Am J Anatomy.

[3] Clevers HC, Bevins CL (2013). Paneth cells: maestros of the small intestinal crypts. Annu Rev Physiol.

[4] Sato T, Vries RG, Snippert HJ, van de Wetering M, Barker N, Stange DE, van Es JH, Abo A, Kujala P, Peters PJ (2009). Single Lgr5 stem cells build crypt–villus structures in vitro without a mesenchymal niche. Nature.

[5] El Aidy S, van den Bogert B, Kleerebezem M (2015). The small intestine microbiota, nutritional modulation and relevance for health. Curr Opin Biotech.

[6] Ostaff MJ, Stange EF, Wehkamp J (2013). Antimicrobial peptides and gut microbiota in homeostasis and pathology. EMBO Mol Med.

[7] Takeda N, Jain R, LeBoeuf MR, Wang Q, Lu MM, Epstein JA (2011). Interconversion between intestinal stem cell populations in distinct niches. Science.

[8] Yu S, Tong K, Zhao Y, Balasubramanian I, Yap GS, Ferraris RP, Bonder EM, Verzi MP, Gao N (2018). Paneth cell multipotency induced by notch activation following injury. Cell Stem Cell.

[9] Smith NR, Davies PS, Silk AD (2012). Wong MH: Epithelial and mesenchymal contribution to the niche: a safeguard for intestinal stem cell homeostasis. Gastroenterology.

[10] Clevers H, Loh KM, Nusse R (2014). An integral program for tissue renewal and regeneration: Wnt signaling and stem cell control. Science.

[11] He XC, Zhang J, Tong WG, Tawfik O, Ross J, Scoville DH, Tian Q, Zeng X, He X, Wiedemann LM (2004). BMP signaling inhibits intestinal stem cell self-renewal through suppression of Wnt-beta-catenin signaling. Nat Genet.

[12] Medema JP, Vermeulen L (2011). Microenvironmental regulation of stem cells in intestinal homeostasis and cancer. Nature.

[13] Samuelson LC (2018). Debate over the identity of an intestinal niche-cell population settled. Nature.

[14] Durand A, Donahue B, Peignon G, Letourneur F, Cagnard N, Slomianny C, Perret C, Shroyer NF, Romagnolo B (2012). Functional intestinal stem cells after Paneth cell ablation induced by the loss of transcription factor Math1 (Atoh1). P Natl Acad Sci USA.

[15] Kim T-H, Escudero S, Shivdasani RA (2012). Intact function of Lgr5 receptor-expressing intestinal stem cells in the absence of Paneth cells. P Natl Acad Sci USA.

[16] Degirmenci B, Valenta T, Dimitrieva S, Hausmann G, Basler K (2018). GLI1-expressing mesenchymal cells form the essential Wnt-secreting niche for colon stem cells. Nature.

[17] Sato T, van Es JH, Snippert HJ, Stange DE, Vries RG, van den Born M, Barker N, Shroyer NF, van de Wetering M, Clevers H (2011). Paneth cells constitute the niche for Lgr5 stem cells in intestinal crypts. Nature.

[18] van Es JH, Wiebrands K, López-Iglesias C, van de Wetering M, Zeinstra L, van den Born M, Korving J, Sasaki N, Peters PJ, van Oudenaarden A (2019). Enteroendocrine and tuft cells support Lgr5 stem cells on Paneth cell depletion. P Natl Acad Sci USA.

[19] Pentinmikko N, Iqbal S, Mana M, Andersson S, Cognetta AB, Suciu RM, Roper J, Luopajärvi K, Markelin E, Gopalakrishnan S (2019). Notum produced by Paneth cells attenuates regeneration of aged intestinal epithelium. Nature.

[20] Farin HF, Van Es JH, Clevers H (2012). Redundant sources of Wnt regulate intestinal stem cells and promote formation of paneth cells. Gastroenterology.

[21] Stzepourginski I, Nigro G, Jacob J-M, Dulauroy S, Sansonetti PJ, Eberl G, Peduto L (2017). CD34+ mesenchymal cells are a major component of the intestinal stem cells niche at homeostasis and after injury. P Natl Acad Sci USA.

[22] Greicius G, Kabiri Z, Sigmundsson K, Liang C, Bunte R, Singh MK, Virshup DM (2018). PDGFRα+ pericryptal stromal cells are the critical source of Wnts and RSPO3 for murine intestinal stem cells in vivo. P Natl Acad Sci USA.

[23] Kabiri Z, Greicius G, Madan B, Biechele S, Zhong Z, Zaribafzadeh H, Edison, Aliyev J, Wu Y, Bunte R (2014). Stroma provides an intestinal stem cell niche in the absence of epithelial Wnts. Development.

[24] McCarthy N, Manieri E, Storm EE, Saadatpour A, Luoma AM, Kapoor VN, Madha S, Gaynor LT, Cox C, Keerthivasan S (2020). Distinct Mesenchymal cell populations generate the essential intestinal BMP signaling gradient. Cell Stem Cell.

[25] Kim J-E, Fei L, Yin W-C, Coquenlorge S, Rao-Bhatia A, Zhang X, Shi SSW, Lee JH, Hahn NA, Rizvi W (2020). Single cell and genetic analyses reveal conserved populations and signaling mechanisms of gastrointestinal stromal niches. Nat Commun.

[26] Roulis M, Kaklamanos A, Schernthanner M, Bielecki P, Zhao J, Kaffe E, Frommelt L-S, Qu R, Knapp MS, Henriques A (2020). Paracrine orchestration of intestinal tumorigenesis by a mesenchymal niche. Nature.

[27] Kinchen J, Chen HH, Parikh K, Antanaviciute A, Jagielowicz M, Fawkner-Corbett D, Ashley N, Cubitt L, Mellado-Gomez E, Attar M (2018). Structural remodeling of the human colonic mesenchyme in inflammatory bowel disease. Cell.

[28] Shoshkes-Carmel M, Wang YJ, Wangensteen KJ, Tóth B, Kondo A, Massasa EE, Itzkovitz S, Kaestner KH (2018). Subepithelial telocytes are an important source of Wnts that supports intestinal crypts. Nature.

[29] Madison BB, McKenna LB, Dolson D, Epstein DJ, Kaestner KH (2009). FoxF1 and FoxL1 link hedgehog signaling and the control of epithelial proliferation in the developing stomach and intestine. J Biol Chem.

[30] Valenta T, Degirmenci B, Moor AE, Herr P, Zimmerli D, Moor MB, Hausmann G, Cantù C, Aguet M, Basler K (2016). Wnt ligands secreted by subepithelial mesenchymal cells are essential for the survival of intestinal stem cells and gut homeostasis. Cell Rep.

[31] Rao DM, Shackleford MT, Bordeaux EK, Sottnik JL, Ferguson RL, Yamamoto TM, Wellberg EA, Bitler BG, Sikora MJ (2019). Wnt family member 4 (WNT4) and WNT3A activate cell-autonomous Wnt signaling independent of porcupine O-acyltransferase or Wnt secretion. J Biol Chem.

[32] Erlenhardt N, Yu H, Abiraman K, Yamasaki T, Wadiche JI, Tomita S, Bredt DS (2016). Porcupine controls hippocampal AMPAR levels, composition, and synaptic transmission. Cell Rep.

[33] Tang S-J, Richards MH, Seaton MS, Wallace J, Al-Harthi L (2014). Porcupine is not required for the production of the majority of Wnts from primary human astrocytes and CD8+ T cells. PLoS ONE.

[34] McCarthy N, Kraiczy J, Shivdasani RA (2020). Cellular and molecular architecture of the intestinal stem cell niche. Nat Cell Biol.

[35] Zhu G, Hu J, Xi R (2021). The cellular niche for intestinal stem cells: a team effort. Cell Reg.

[36] Sailaja BS, He XC, Li L (2016). The regulatory niche of intestinal stem cells. J Physiol.

[37] Worthley Daniel L, Churchill M, Compton Jocelyn T, Tailor Y, Rao M, Si Y, Levin D, Schwartz Matthew G, Uygur A, Hayakawa Y (2015). Gremlin 1 identifies a skeletal stem cell with bone, cartilage, and reticular stromal potential. Cell.

[38] Yu K, Xu J, Liu Z, Sosic D, Shao J, Olson EN, Towler DA, Ornitz DM (2003). Conditional inactivation of FGF receptor 2 reveals an essential role for FGF signaling in the regulation of osteoblast function and bone growth. Development.

[39] Greenbaum A (2013). Hsu Y-MS, Day RB, Schuettpelz LG, Christopher MJ, Borgerding JN, Nagasawa T, Link DC: CXCL12 in early mesenchymal progenitors is required for haematopoietic stem-cell maintenance. Nature.

[40] Méndez-Ferrer S, Michurina TV, Ferraro F, Mazloom AR, MacArthur BD, Lira SA, Scadden DT (2010). Ma’ayan A, Enikolopov GN, Frenette PS: Mesenchymal and haematopoietic stem cells form a unique bone marrow niche. Nature.

[41] Geske MJ, Zhang X, Patel KK, Ornitz DM, Stappenbeck TS (2008). Fgf9 signaling regulates small intestinal elongation and mesenchymal development. Development.

[42] Horiguchi H, Endo M, Kawane K, Kadomatsu T, Terada K, Morinaga J, Araki K, Miyata K, Oike Y (2017). ANGPTL2 expression in the intestinal stem cell niche controls epithelial regeneration and homeostasis. EMBO J.

[43] San Roman Adrianna K, JayewickremeChenura D, Murtaugh LC, Shivdasani Ramesh A (2014). Wnt secretion from epithelial cells and subepithelial myofibroblasts is not required in the mouse intestinal stem cell niche in vivo. Stem Cell Rep.

[44] Buch T, Heppner FL, Tertilt C, Heinen TJ, Kremer M, Wunderlich FT, Jung S, Waisman A (2005). A Cre-inducible diphtheria toxin receptor mediates cell lineage ablation after toxin administration. Nat Method.

[45] Gao L, Yu Q, Zhang H, Wang Z, Zhang T, Xiang J, Yu S, Zhang S, Wu H, Xu Y (2021). A resident stromal cell population actively restrains innate immune response in the propagation phase of colitis pathogenesis in mice. Sci Transl Med.

[46] He D, Wu H, Xiang J, Ruan X, Peng P, Ruan Y, Chen Y-G, Wang Y, Yu Q, Zhang H (2020). Gut stem cell aging is driven by mTORC1 via a p38 MAPK-p53 pathway. Nat Commun.

[47] Bänziger C, Soldini D, Schütt C, Zipperlen P, Hausmann G, Basler K (2006). Wntless, a conserved membrane protein dedicated to the secretion of wnt proteins from signaling cells. Cell.

[48] Walton KD, Kolterud Å, Czerwinski MJ, Bell MJ, Prakash A, Kushwaha J, Grosse AS, Schnell S, Gumucio DL (2012). Hedgehog-responsive mesenchymal clusters direct patterning and emergence of intestinal villi. P Natl Acad Sci USA.

[49] Chin Alana M, Tsai Y-H, Finkbeiner Stacy R, Nagy Melinda S, Walker Emily M, Ethen Nicole J, Williams Bart O, Battle Michele A, Spence Jason R (2016). A dynamic WNT/β-CATENIN signaling environment leads to WNT-Independent and WNT-dependent proliferation of embryonic intestinal progenitor cells. Stem Cell Rep.

[50] Sumigray KD, Terwilliger M, Lechler T (2018). Morphogenesis and compartmentalization of the intestinal crypt. Dev Cell.

[51] Adolph TE, Tomczak MF, Niederreiter L, Ko H-J, Böck J, Martinez-Naves E, Glickman JN, Tschurtschenthaler M, Hartwig J, Hosomi S (2013). Paneth cells as a site of origin for intestinal inflammation. Nature.

[52] Karin M, Clevers H (2016). Reparative inflammation takes charge of tissue regeneration. Nature.

[53] Saha S, Aranda E, Hayakawa Y, Bhanja P, Atay S, Brodin NP, Li J, Asfaha S, Liu L, Tailor Y (2016). Macrophage-derived extracellular vesicle-packaged WNTs rescue intestinal stem cells and enhance survival after radiation injury. Nat Commun.

[54] Schmitt M, Schewe M, Sacchetti A, Feijtel D, van de Geer WS, Teeuwssen M, Sleddens HF, Joosten R, van Royen ME, van de Werken HJG (2018). Paneth cells respond to inflammation and contribute to tissue regeneration by acquiring stem-like features through SCF/c-Kit signaling. Cell Rep.

[55] McCarthy N, Manieri E, Storm EE, Saadatpour A et al. Distinct Mesenchymal Cell Populations Generate the Essential Intestinal BMP Signaling Gradient. 2020. NCBI; https://www.ncbi.nlm.nih.gov/geo/query/acc.cgi?acc=GSM4196131. 10.1016/j.stem.2020.01.008 PMC7412576 32084389

[56] McCarthy N, Manieri E, Storm EE, Saadatpour A et al. Distinct Mesenchymal Cell Populations Generate 56.the Essential Intestinal BMP Signaling Gradient. 2020. NCBI; https://www.ncbi.nlm.nih.gov/geo/query/acc.cgi?acc=GSM4196132. 10.1016/j.stem.2020.01.008 PMC7412576 32084389

