# Supplementary material for: A stromal lineage maintains crypt structure and villus homeostasis in the intestinal stem cell niche
Source: BMC Biol. 2023 Aug 8;21:169. doi: 10.1186/s12915-023-01667-2 (PMC10408166; doi:10.1186/s12915-023-01667-2)
Supplement: Supplementary file 1 — Additional file 1: Fig. S1. tSNE analysis of intestinal cells of mice. Fig. S2. The intestinal Twist2 and Gli1 lineage cells expressed different surface markers. Fig. S3. Effects of depletion of Twist2, Prrx1, or Nestin lineage cells on the villi. Fig. S4. Depletion of Twist2 lineage cells impaired crypt regeneration. Fig. S5. tSNE analysis of Wnt and Rspo expression in intestinal cells. Fig. S6. Ablation of one Wls allele led to a decrease in β-Catenin in epithelial cells. Fig. S7. Effects of deletion of one copy of Wls in Prrx1 or Acta2 lineages on villus homeostasis. Fig. S8. Deletion of one Wls allele in Prrx1 lineage cells did not affect villus regeneration. Fig. S9. IR induced expression of inflammation-related cytokines and AMPs. Fig. S10. LPS-induced inflammation impaired ISC regeneration. Table S1. Primer sequences used for quantitative PCR. [file 12915_2023_1667_MOESM1_ESM.docx]

**Supplementary Information for:**

**A stromal lineage acts an intestinal stem cell niche by maintaining the crypt structure and restraining gut bacteria**

Jinnan Xiang^1*^, Jigang Guo^1*^, Shaoyang Zhang^1^, Hongguang Wu^1^, Ye-Guang Chen^2^, Junping Wang^3^, Baojie Li ^1,4^, Huijuan Liu^1,4^

**Supplementary Figure legends**

**
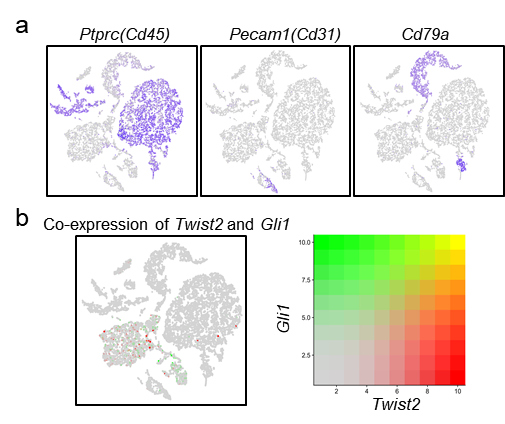
**

**Fig. S1. tSNE analysis of intestinal cells of mice.**

**a.** A scRNA-seq dataset (PMID 32084389) was used to analyzed the expression of *Cd45*, *Cd31*, and *Cd79a*, which were used to indicate immune cells, endothelial cells, and plasma cells, respectively.

**b**. Comparison of the expression of *Gli1* and *Twist2* in *Pdgfra^low^* stromal cells and trophocytes.

**
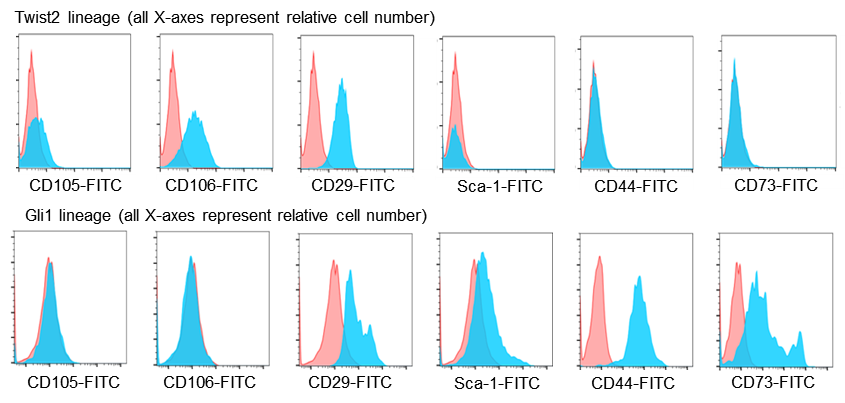
Fig. S2. The intestinal *Twist2* and *Gli1* lineage cells expressed different surface markers.**

Flow cytometry analysis of the MSC-related surface markers expressed by intestinal *Twist2* or *Gli1* lineage stromal cells. The intestinal stromal cells were isolated from *Twist2-Cre; tdTomato* mice and *Gli1-Cre/ERT; ROSA-tdTomato* mice (2 months after 3 doses TAM injection), incubated with different surface marker antibodies, and analyzed with flow cytometry. Only tdTomato-labeled cells were analyzed. X-axis represents the fluorescence intensity of indicated FACS antibodies while Y-axis represents relative cell numbers.

**
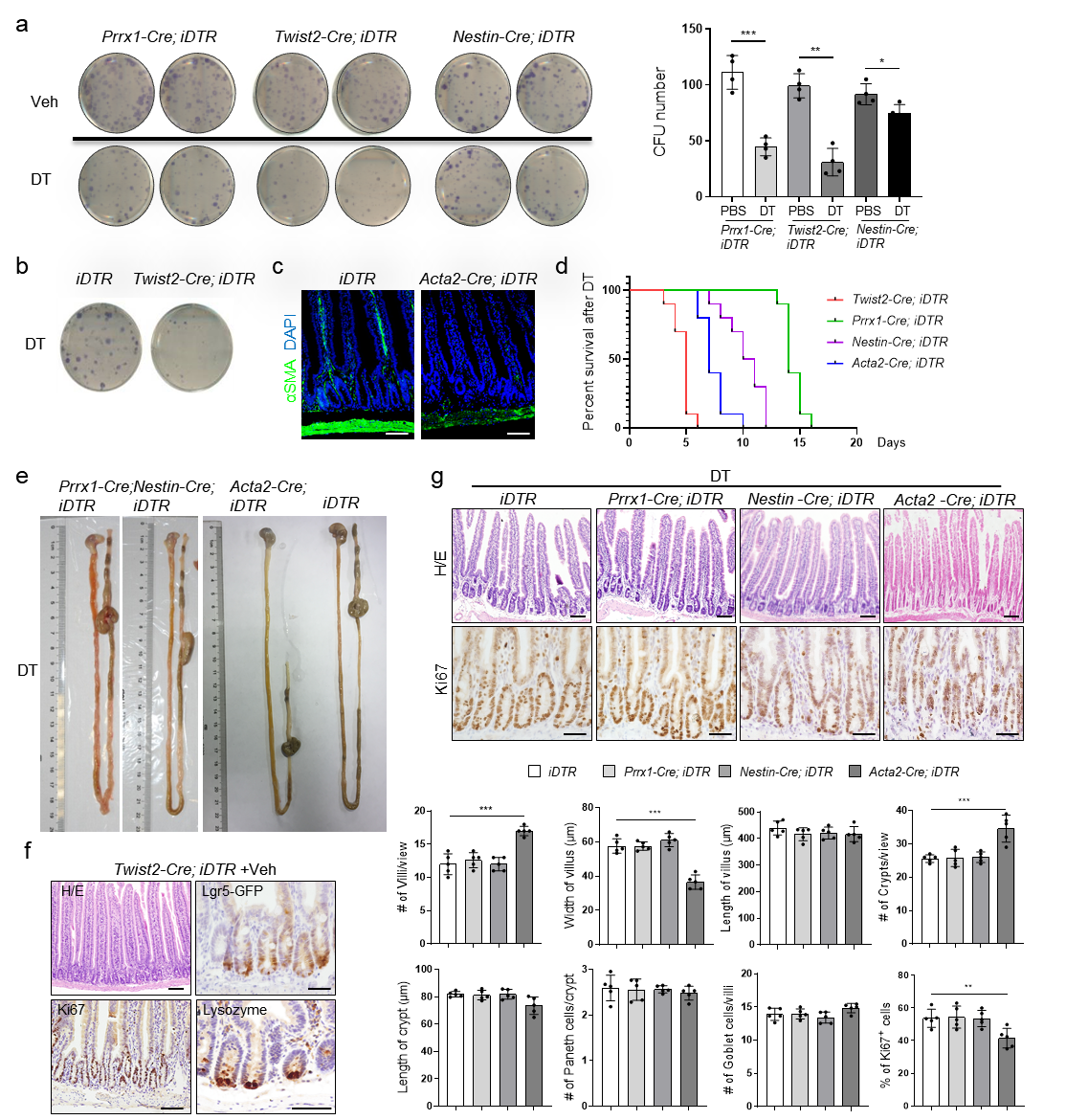
Fig. S3. Effects of depletion of *Twist2*, *Prrx1*, or *Nestin* lineage cells on the villi.**

**a**. The reduction of F-CFU in *Twist2-Cre; iDTR, Prrx1-Cre; iDTR*, and *Nestin-Cre; iDTR* mouse lines after DT administration. Since iDTR is simian HB-EGF, we used PBS-treated *Cre-iDTR* mouse lines as controls. Right panel: quantitation data. *P<0.05, **P<0.01, and ***P<0.001. N=4.

**b**. DT-treated *iDTR* mice was used as a control for DT-treated *Twist2-Cre; iDTR* mice.

**c**. Immunostaining of αSMA showed that *Acta2* lineage cells were reduced in the intestines of *Acta2-Cre; iDTR* mice. Bar: 50 μm.

**d**. The survival time of the four mouse lines after DT administration. N=10.

**e**. The effects of depletion of *Prrx1*, *Nestin*, or *Acta2* lineage cells on the morphology of the GI tracts.

**f**. PBS-treated *Twist2-Cre; iDTR* mice was used as a control for *Twist2-Cre; iDTR* mice treated with DT (Fig. 2b). Bar: 50 μm.

**g**. H/E and Ki67 staining of the intestine sections of *Prrx1-Cre; iDTR* mice, *Nestin-Cre; iDTR* mice, and *Acta2-Cre; iDTR* mice. Bar: 50 μm. Bottom panels: quantitation data. *P<0.05, **P<0.01 and ***P<0.001. N=5.

**
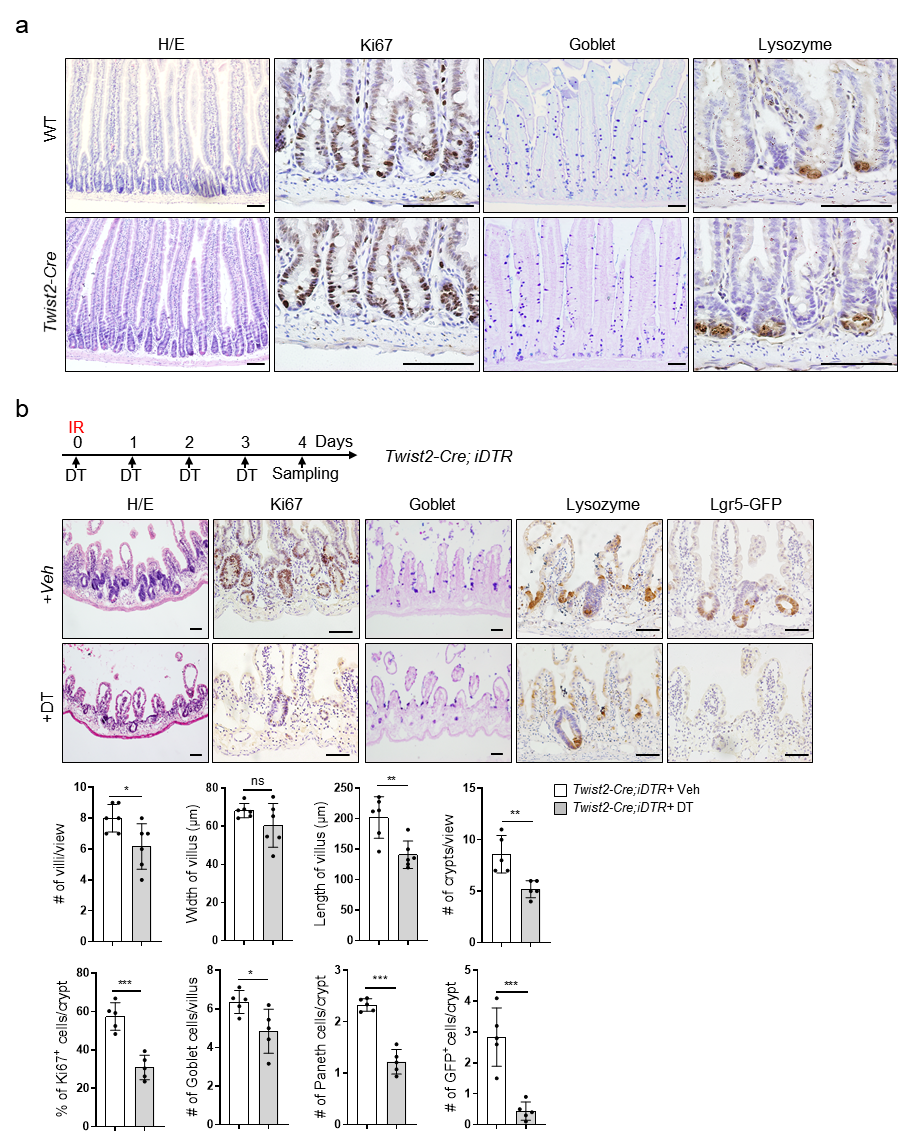
Fig. S4. Depletion of *Twist2* lineage cells impaired crypt regeneration.**

**a**. Adult *Twist2-Cre* mice showed normal villus structure and normal cell proliferation and differentiation of goblet and Paneth cells.

**b**. H/E, Ki67, goblet, lysozyme, and GFP staining of normal and *Twist2* cell-depleted mouse intestinal villi 4 days after IR. Bar: 50 μm. Bottom panels: quantitation data. *P<0.05, **P<0.01, and ***P<0.001. N=6 (# of villi/view, Width of villus and Length of villus). Others N=5.

**
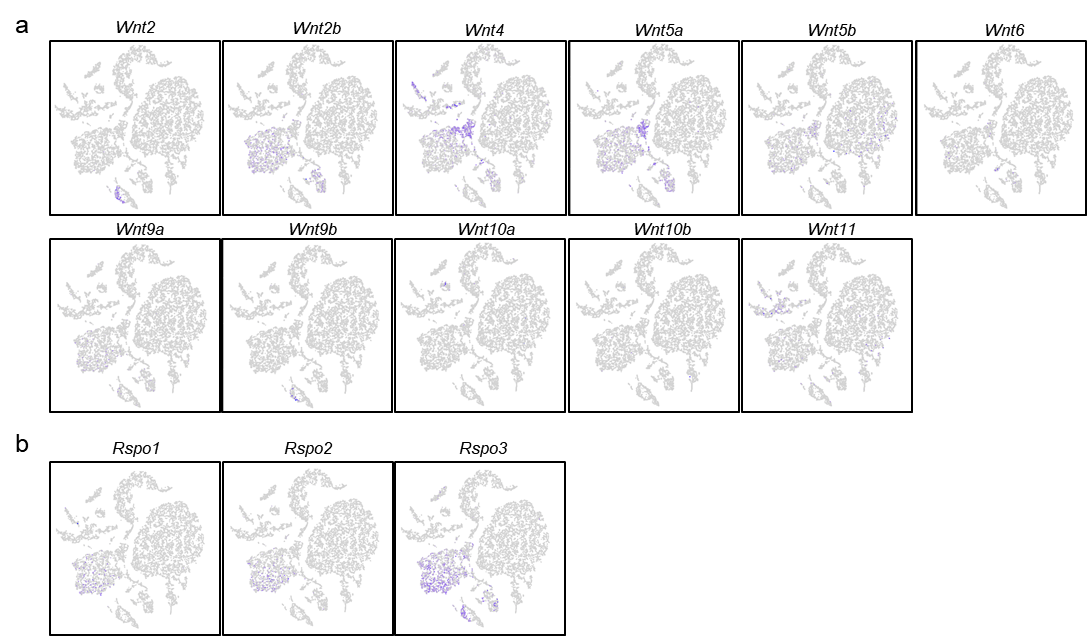
Fig. S5. tSNE analysis of *Wnt* and *Rspo* expression in intestinal cells.**

**a.** A scRNA-seq dataset (PMID 32084389) was used to analyzed the expression of various *Wnt* molecules in the intestinal cells of adult mice.

**b**. A scRNA-seq dataset (PMID 32084389) was used to analyzed the expression of various *Rspo* molecules in the intestinal cells of adult mice.

**
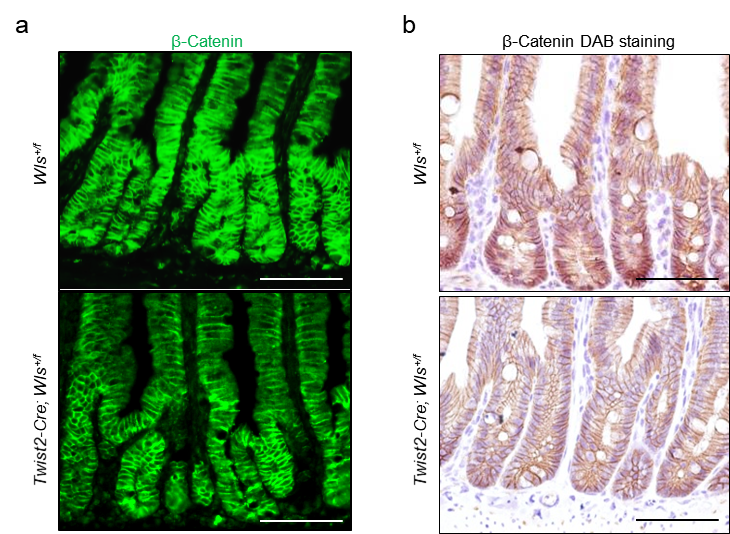
**

**Fig. S6. Ablation of one *Wls* allele led to a decrease in β-Catenin in epithelial cells.**

**a**. Immunofluorescent staining for β-Catenin in the intestinal villi of control and *Twist2-Cre;Wls^+/f^* mice. Bar: 50 μm.

**b.** Immunohistochemical staining for β-Catenin in the intestinal villi of control and *Twist2-Cre;Wls^+/f^* mice. Bar: 50 μm.

**
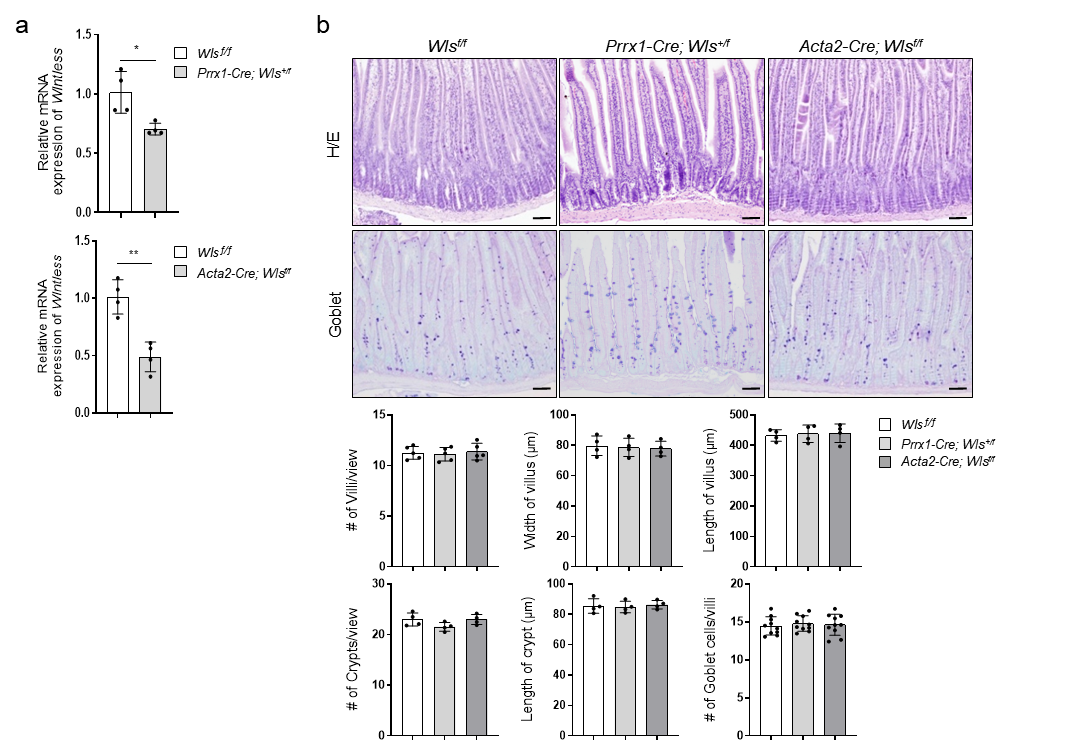
Fig. S7. Effects of deletion of one copy of *Wls* in *Prrx1* or *Acta2^+^* lineages on villus homeostasis.**

**a**. Quantitative PCR results showed a decrease in the mRNA levels of *Wls* in the stromal cells (Tomato^+^) of the small intestine of *Prrx1-Cre;Tomato;Wls^+/f^* mice or *Acta2-Cre;Tomato;Wls^f/f^* mice compared to control mice. N=4.

**b**. Ablation of one copy of *Wls* in *Prrx1* lineage cells or ablation of two copies of *Wls* in *Acta2* lineage cells did not affect villus homeostasis. Bar: 50 μm. Bottom panels: Quantitation data. N=4.

**
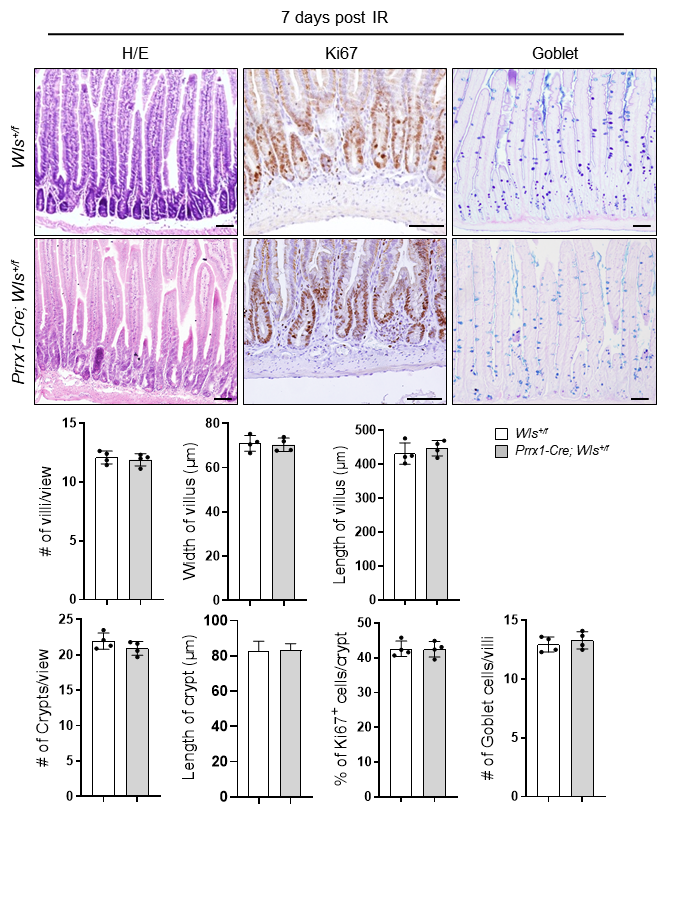
**

**Fig. S8. Deletion of one *Wls* allele in *Prrx1* lineage cells did not affect villus regeneration.**

*Prrx1-Cre; Wls^+/f^* mice showed normal ISC proliferation or differentiation during villus regeneration 7 days after IR. Bar: 50 μm. Bottom panels: quantitation data. N=4.

**
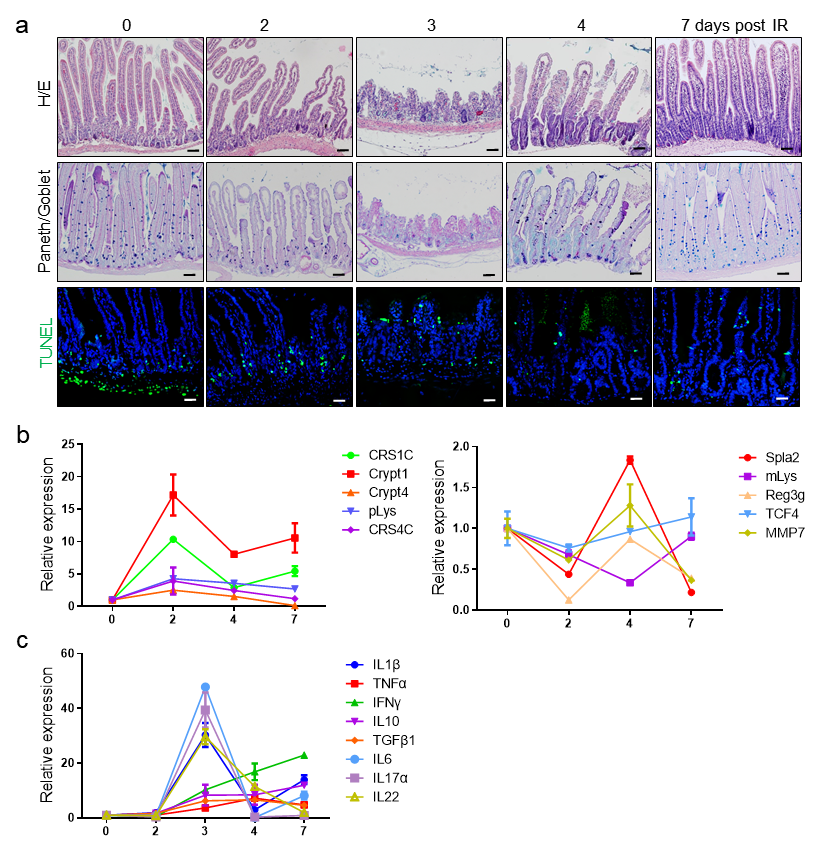
**

**Fig. S9. IR induced expression of inflammation-related cytokines and AMPs.**

**a**. H/E, Paneth/goblet, and TUNEL staining of intestine sections of wild type mice that were radiated and sacrificed at different time. Bar: 50 μm.

**b**. Quantitative PCR results showed the change of defensin/cryptidin molecules in small intestines after IR. Total RNA was isolated from the radiated mouse small intestines and used for reverse transcription. qPCR was carried out to determine the mRNA levels of these molecules. The basal levels of no radiated mouse small intestines mRNA were set at 1.0. N=3.

**c**. Quantitative PCR results showed the change of inflammation-related cytokines in small intestines after IR. Total RNA was isolated from the radiated mouse small intestines and used for reverse transcription. Realtime PCR was carried out to determine the mRNA levels of these cytokines. The basal levels of no radiated mouse small intestines mRNA were set at 1.0. N=3.

**
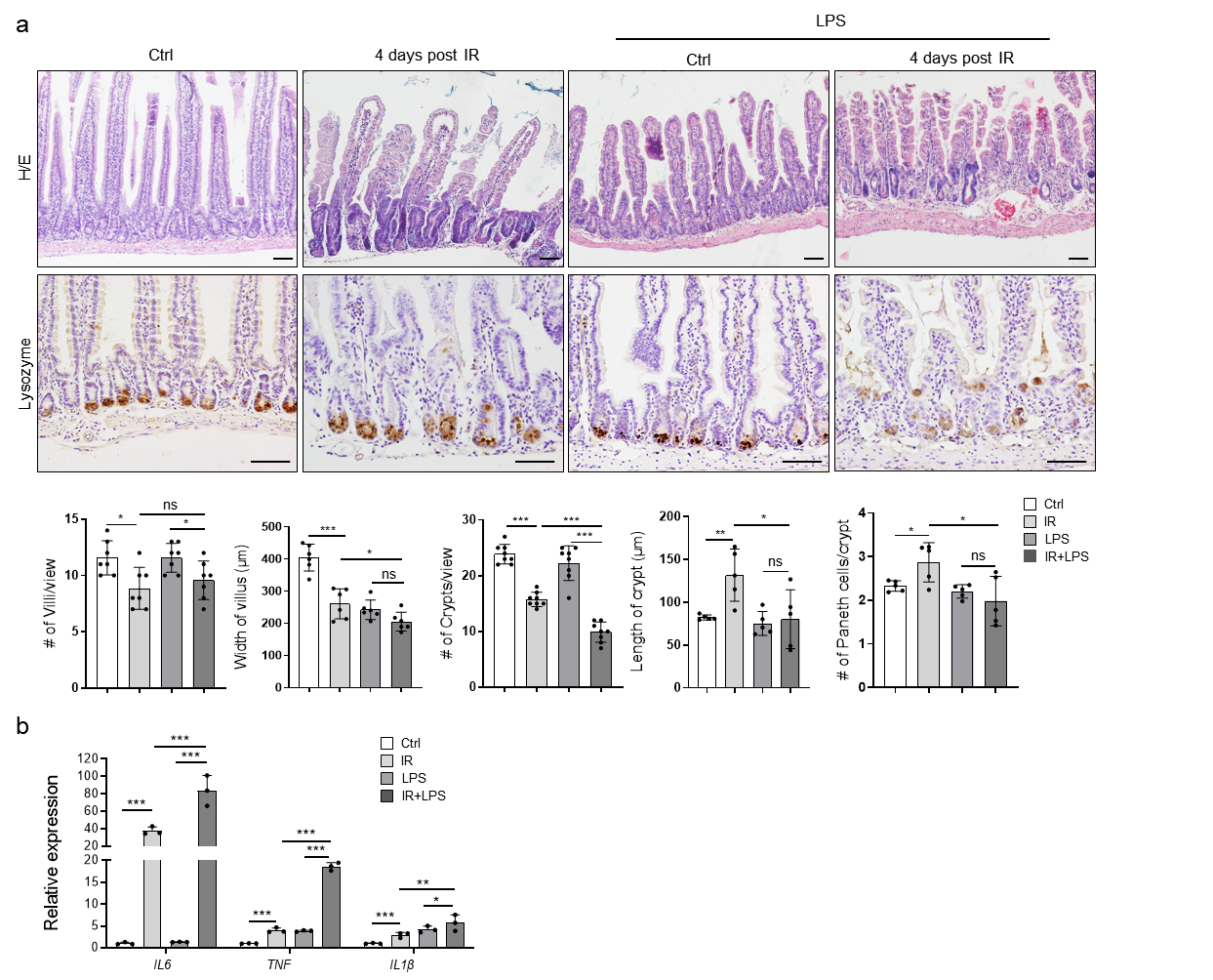
Figure S10.** **LPS-induced inflammation impaired ISC regeneration.**

**a**. H/E and Paneth cell staining of intestine sections of LPS-treated mice that were radiated and sacrificed after 4 days. Bar: 50 μm. Bottom panels: quantitation data. *P<0.05, **P<0.01, and ***P<0.001. N=8 (# of crypts/view). N=7 (# of villi/view). N=6 (Width of villus). N=5 (Length of crypt and # of Paneth cells/crypt).

**b.** LPS augmented the expression of inflammatory cytokines in the intestines. The levels of each cytokine in control group were set at 1.0. *P<0.05, **P<0.01, and ***P<0.001. N=3.

**
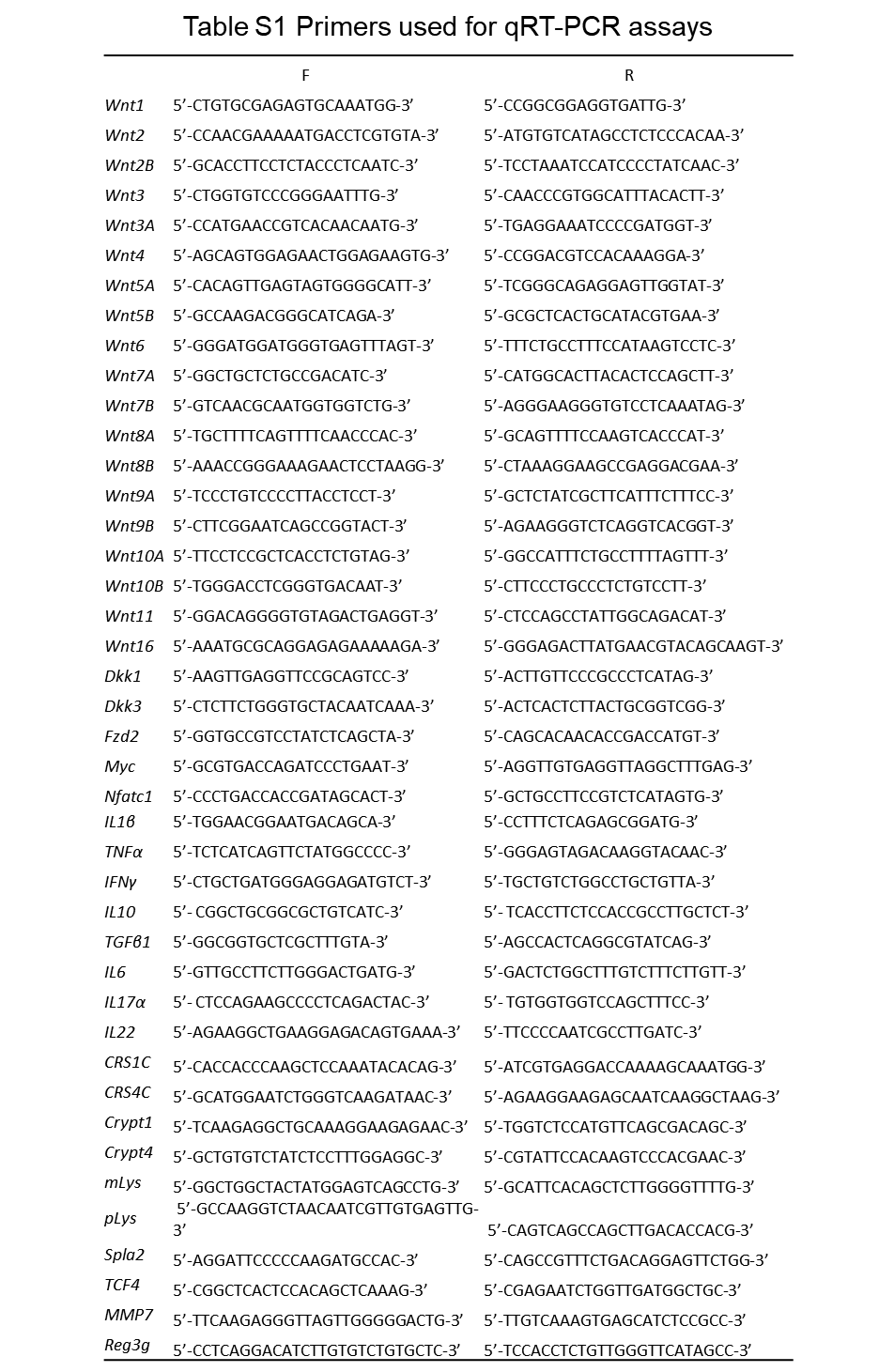
Supplementary Table S1.** Primer sequences used for quantitative PCR.
